# The Dark Side of the Mushroom Spring Microbial Mat: Life in the Shadow of Chlorophototrophs. I. Microbial Diversity Based on 16S rRNA Gene Amplicons and Metagenomic Sequencing

**DOI:** 10.3389/fmicb.2016.00919

**Published:** 2016-06-17

**Authors:** Vera Thiel, Jason M. Wood, Millie T. Olsen, Marcus Tank, Christian G. Klatt, David M. Ward, Donald A. Bryant

**Affiliations:** ^1^Department of Biochemistry and Molecular Biology, The Pennsylvania State UniversityUniversity Park, PA, USA; ^2^Department of Land Resources and Environmental Sciences, Montana State UniversityBozeman, MT, USA; ^3^Agricultural Research Service, United States Department of Agriculture, University of MinnesotaSaint Paul, MN, USA; ^4^Department of Chemistry and Biochemistry, Montana State UniversityBozeman, MT, USA

**Keywords:** hot spring, microbial community, microbial diversity, extreme environments, phototrophic bacteria

## Abstract

Microbial-mat communities in the effluent channels of Octopus and Mushroom Springs within the Lower Geyser Basin at Yellowstone National Park have been studied for nearly 50 years. The emphasis has mostly focused on the chlorophototrophic bacterial organisms of the phyla *Cyanobacteria* and *Chloroflexi*. In contrast, the diversity and metabolic functions of the heterotrophic community in the microoxic/anoxic region of the mat are not well understood. In this study we analyzed the orange-colored undermat of the microbial community of Mushroom Spring using metagenomic and rRNA-amplicon (iTag) analyses. Our analyses disclosed a highly diverse community exhibiting a high degree of unevenness, strongly dominated by a single taxon, the filamentous anoxygenic phototroph, *Roseiflexus* spp. The second most abundant organisms belonged to the *Thermotogae*, which have been hypothesized to be a major source of H_2_ from fermentation that could enable photomixotrophic metabolism by *Chloroflexus* and *Roseiflexus* spp. Other abundant organisms include two members of the *Armatimonadetes* (OP10); *Thermocrinis* sp.; and phototrophic and heterotrophic members of the *Chloroflexi*. Further, an *Atribacteria* (OP9/JS1) member; a sulfate-reducing *Thermodesulfovibrio* sp.; a *Planctomycetes* member; a member of the EM3 group tentatively affiliated with the *Thermotogae*, as well as a putative member of the *Arminicenantes* (OP8) represented ≥1% of the reads. *Archaea* were not abundant in the iTag analysis, and no metagenomic bin representing an archaeon was identified. A high microdiversity of 16S rRNA gene sequences was identified for the dominant taxon, *Roseiflexus* spp. Previous studies demonstrated that highly similar *Synechococcus* variants in the upper layer of the mats represent ecological species populations with specific ecological adaptations. This study suggests that similar putative ecotypes specifically adapted to different niches occur within the undermat community, particularly for *Roseiflexus* spp.

## Introduction

Microbial mat communities inhabiting the effluent channels of Octopus and Mushroom Springs within the Lower Geyser Basin at Yellowstone National Park (YNP) have been studied for nearly 50 years (Brock, 1967; Ward et al., 2012). In these studies, the chlorophototrophic bacterial populations, i.e., chlorophyll-based phototrophs including members of the *Cyanobacteria, Chloroflexi* and the newly discovered *Chloracidobacterium (Cab.) thermophilum* and “*Candidatus* Thermochlorobacter (Tcb.) aerophilum,” have generally been the main focus (Bauld and Brock, 1973; Nold and Ward, 1996; Bryant et al., 2007; van der Meer et al., 2007; Steunou et al., 2008; Becraft et al., 2011; Klatt et al., 2011, 2013b; Liu et al., 2011, 2012; Tank and Bryant, 2015a,b). In contrast, the diversity and metabolic functions of the heterotrophic community in the microoxic/anoxic region of the mat are not well understood.

**Figure 1:**
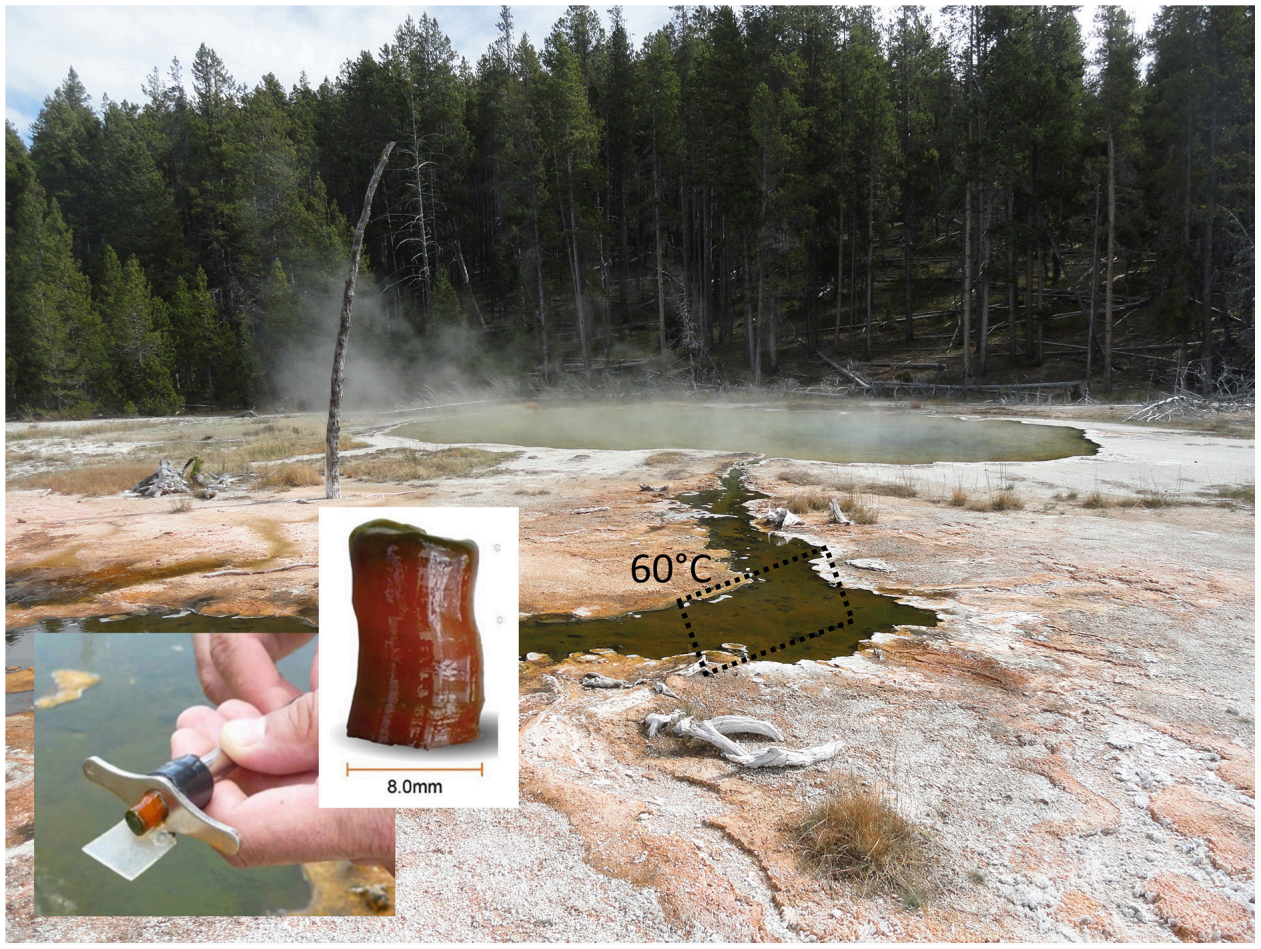
**Sampling site at Mushroom Spring, Yellowstone National Park, and microbial mat core (adapted from Kim et al., 2015)**.

Using cultivation-based methods, early studies focused on the dominant *Cyanobacteria* and phototrophic *Chloroflexi* (Bauld and Brock, 1973; Bateson and Ward, 1988). Over time, these studies were extended by a variety of molecular methods with increasing molecular resolution. A pioneering molecular study targeting 16S rRNA gene sequences directly indicated a greater diversity of uncultivated bacteria in the mat than previously realized (Ward et al., 1990). However, only recently have metagenomic (Klatt et al., 2011), metatranscriptomic (Liu et al., 2011, 2012; Klatt et al., 2013b) and metametabolomic (Kim et al., 2015) analyses led to a holistic overview, in terms of the organisms present and their functional potentials, of the major taxa inhabiting the upper 2 mm of the 60–65°C regions of the Mushroom Spring microbial mats (Figure 1). The microbial community of the upper green mat layer contains eight dominant bacterial populations, of which six are chlorophototrophs (Klatt et al., 2011). Oxygenic cyanobacteria from the genus *Synechococcus* have been shown to be the predominant primary producers in these communities by *in situ* studies of bicarbonate fixation and nitrogen fixation (Steunou et al., 2008) using stable and radioactive isotopes (Bateson and Ward, 1988; Nübel et al., 2002; van der Meer et al., 2007). In addition, anoxygenic photoheterotrophic members of the *Roseiflexus* spp. have been suggested to perform inorganic carbon fixation (van der Meer et al., 2003, 2005, 2007, 2010; Klatt et al., 2007, 2013b). *Synechococcus* spp. fix CO_2_ and synthesize and excrete metabolites that are then consumed by (photo)heterotrophic members of the community, including several members of the *Chloroflexi*, and presumably *Roseiflexus* spp. (Anderson et al., 1987; Bateson and Ward, 1988; Kim et al., 2015). Collectively, cyanobacteria and *Roseiflexus* spp. account for the majority of the biomass of the upper 0–2 mm portion of the mat community. Two additional members of the phylum *Chloroflexi, Chloroflexus* sp. and an apparently phototrophic, “*Anaerolineae*-like” organism (“*Ca*. Roseilinea gracile”; Tank et al., in press), as well as two recently discovered aerobic/microaerophilic, anoxygenic photoheterotrophs, *Cab. thermophilum* (Bryant et al., 2007; Garcia Costas et al., 2012a,b; Tank and Bryant, 2015a,b) and “*Ca*. Tcb. aerophilum” (Liu et al., 2012), also occur in the upper photic layer of the mat.

Early studies on the processes and organisms involved in aerobic and anaerobic decomposition of the mat have been discussed in a review by Ward et al. (1992; and earlier papers cited therein). Since the discovery of the aerobic heterotroph *Thermus aquaticus* (Brock and Freeze, 1969) many aerobic (e.g., *Thermomicrobium roseum;* Jackson et al., 1973) and anaerobic fermentative and sulfate-reducing bacteria were cultivated from these mats (e.g., *Bacillus stearothermophilis, Thermoanaerobium brockii, Thermoanaerobacter ethanolicus, Thermodesulfotobacterium commune;* see Ward et al., 1992 for primary references). Many of the latter were sought with the hope that thermophiles would be useful for biofuel production. However, critical review indicated that most of these isolates had not been cultivated from highly diluted mat samples, and thus their importance to the community remained unknown (see Ward et al., 1998). Indeed, with one exception, *Thermomicrobium roseum* (Wu et al., 2009), the genomes of these organisms did not recruit reads with high identity values from metagenomic analysis of the upper mat layer (Klatt et al., 2011). Only two low-abundance, unidentified heterotrophic bacteria lacking the genes needed to synthesize chlorophyll (Chl) were detected in the upper mat community represented by metagenomic bins (Klatt et al., 2011). Nevertheless, heterotrophs, together with the photoheterotrophic and photomixotrophic community members, can be considered potential consumers of metabolites produced by cyanobacteria and possibly other mat inhabitants. In more recent years, the activity and diversity of sulfate-reducing bacteria of the microbial mats have been more intensively studied. Dillon et al. (2007) showed that an active sulfur cycle occurs in the mat community despite very low sulfate concentrations. The highest rates of sulfate respiration were reportedly associated with *Thermodesulfovibrio*-like organisms and were measured close to the surface of the mat late in the day when photosynthetic oxygen production had ceased. Additionally, methane production has been detected in numerous alkaline siliceous hot spring microbial mats in YNP (Ward, 1978; Sandbeck and Ward, 1981, 1982). Methanogenic archaea (~10^7^ to 10^8^ ml^−1^) have been enumerated in small cores of Octopus Spring mats, which in combination with the detection of low levels of archaeal lipids, suggests that methanogenesis occurred *in situ* in those mats (Ward, 1978; Sandbeck and Ward, 1981; Ward et al., 1985). The relative rarity of these organisms compared to *Synechococcus* (on the order of 1% or less) suggests that these terminal anaerobes receive little of the energy recycled during decomposition of the mat (Ward et al., 1989).

The first revolution of molecular microbial ecology enabled the study of uncultured bacterial diversity through amplification, sequencing and phylogenetic analysis of ribosomal RNA genes (Olsen et al., 1986; Ward et al., 1990; Amann et al., 1995; Hugenholtz and Pace, 1996; Hugenholtz et al., 1998a; Pace, 2009). Through such studies, our perspective on microbial diversity has increased enormously over the past three decades, and the impact of culture-independent studies on the emerging view of bacterial diversity cannot be overstated (Hugenholtz et al., 1998a). Ward and coworkers reported the presence of a number of uncultured bacterial lineages in their first molecular microbial diversity study of the mat community of Octopus Spring (Ward et al., 1990). Over the course of the past 25 years, several of those initially unidentified ribosomal RNA sequences have been associated with chlorophototrophic mat members (OS-A and B with *Synechococcus* spp., OS-C with *Roseiflexus* sp., OS-D with *Cab. thermophilum*, and OS-E with “*Ca*. Tcb. aerophilum”), whereas many others (OS-F, OS-G, OS-H, OS-K, OS-L, OS-M, OS-N, OS-R) still have not been identified and were not detected in the metagenome of the upper green layer (Klatt et al., 2011).

“Red-layer” communities, which may often be “orange” in color as is the case for the mats of Mushroom Spring, have been shown to contain novel chlorophototrophs (Boomer et al., 2000, 2002), whose pigments exhibit unusual *in vivo* absorption spectra (Boomer et al., 2000), but these communities have not yet been studied in detail. As part of a comparative study of YNP hot spring microbial mat communities, a 45-Mbp metagenome based on Sanger sequencing revealed some initial insights into the composition of the undermat microbial community of Mushroom Spring (Klatt et al., 2013a). Compared to the upper green layer, fewer *Synechococcus* spp., a greater number of *Roseiflexus* spp., and several presumed anaerobic or fermentative organisms within the *Bacteroidetes* and *Thermodesulfobacteria* were identified. The undermat community contained a *Thermotoga*-like population as well as several low G+C organisms that could not be characterized (Klatt et al., 2013a). Low coverage and a small number of long scaffolds above the threshold used in most clustering analyses (>10 kb) limited the application of metagenomic binning approaches (Klatt et al., 2013a) and indicated that additional studies with much deeper sequencing would be needed to define the undermat community.

The overall goal of this research is to investigate the complete microbial mat community at Mushroom Spring and to develop a comprehensive understanding of the microbial ecology of the microbial mats of this hot spring. The specific objectives of this study were to analyze the orange-colored undermat community, to identify those organisms that are present, and to facilitate an active integration of these mostly heterotrophic members into models of the mat community. This paper describes the composition and diversity of the Mushroom Spring undermat community based on rRNA-amplicon (iTag) and deep metagenomic sequencing analyses, with an initial focus on the identity and taxonomic diversity of the community members. A description of the metabolic potential and putative interactions, including a metabolic description of the entire microbial mat community, will be published separately.

## Materials and methods

The samples were collected on August 10th, 2011 from a chlorophototrophic microbial mat in an effluent channel of the siliceous and slightly alkaline Mushroom Spring in YNP, WY (USA). The samples were collected using a #4 cork borer at a site where the water above the mat was 60°C (Figure 1). The microbial mat is made up of an upper green layer (1–2 mm thick), which mainly consists of different chlorophototrophic bacteria, and an orange-colored undermat layer (Figure 1). Genomic DNA was extracted from the orange-colored undermat layer (~3–5 mm depth; DNA from below this level was too degraded to analyze). The metagenome as well as 16S rRNA gene PCR amplicons were sequenced at the DOE Joint Genome Institute (JGI) using HiSeq and MiSeq Illumina technologies. The iTtag sequences were analyzed at two different identity levels. All reads were clustered into operational taxonomic units (OTUs) with 97% sequence identity cutoff by using USEARCH, but they were also analyzed after dereplication (i.e., clustered by 100% nt identity, see Supplementary Materials). RDP Classifier (Wang et al., 2007; Cole et al., 2009), BLAST searches (Altschul et al., 1990) and phylogenetic analyses (Ludwig et al., 2004) were used to identify sequences. Microdiversity was assessed using the number of highly abundant dereplicated sequences, and the “oligotyping pipeline” (http://merenlab.org/projects/oligotyping/). HiSeq metagenomic reads were assembled and then clustered into bins by oligonucleotide frequency pattern analyses using ESOM (Dick et al., 2009). Metagenomic bins were treated as partial genomes of single taxa and were taxonomically affiliated using Amphoranet (http://pitgroup.org/amphoranet/, Kerepesi et al., 2014) to assess the phylogenetic marker genes present in each bin. Detailed descriptions of the methods for DNA extraction, library construction, sequencing, and data analyses are found in the Supplementary Materials.

## Results

**Figure 2:**
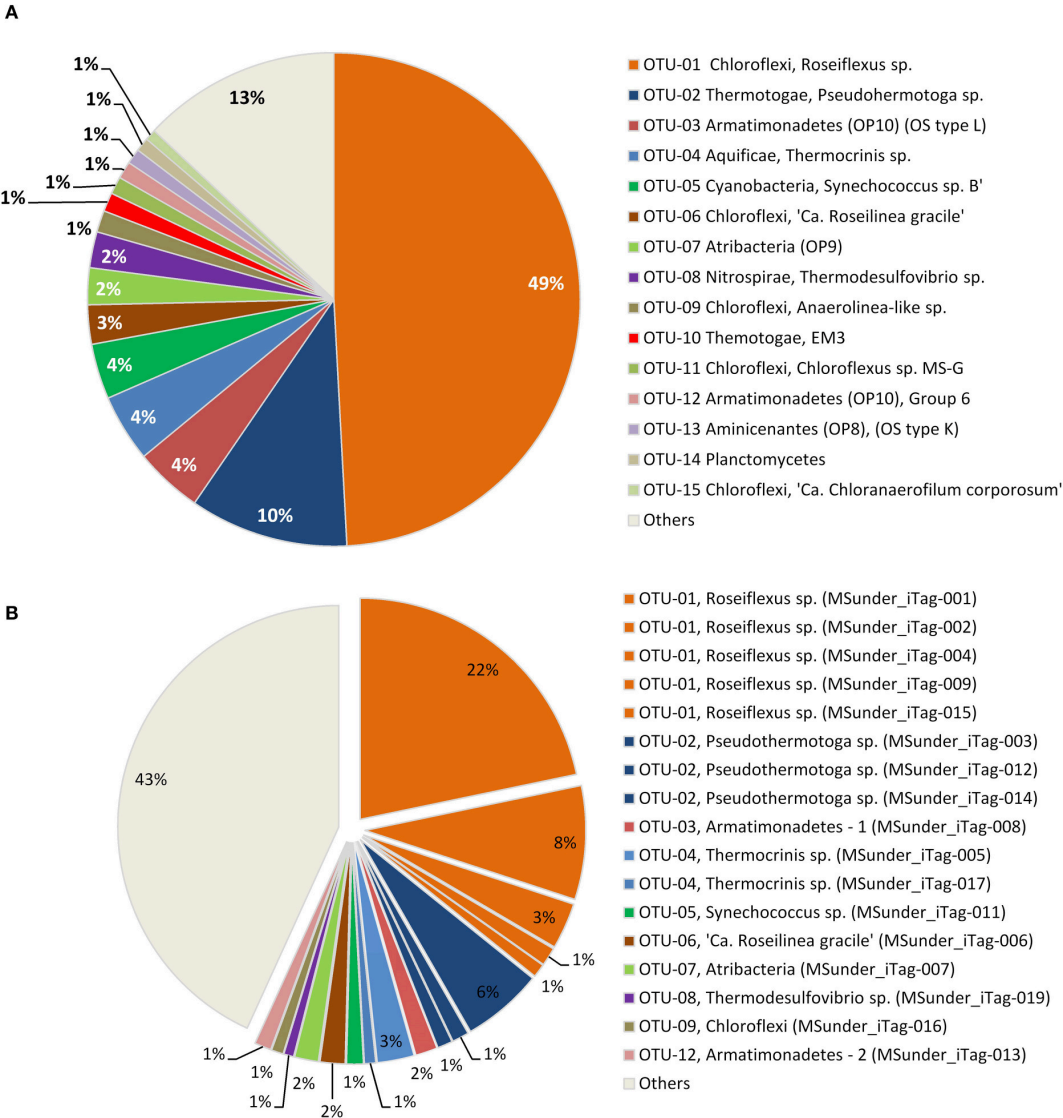
**Relative abundance of (A) the 15 most abundant 97% OTUs, and (B) the 17 most abundant dereplicated iTag sequences in the Mushroom Spring undermat 16S rRNA gene amplicon (iTag) analysis**. All less abundant OTUs (<1,000 reads each) are shown combined as “Others.”

**Figure 3:**
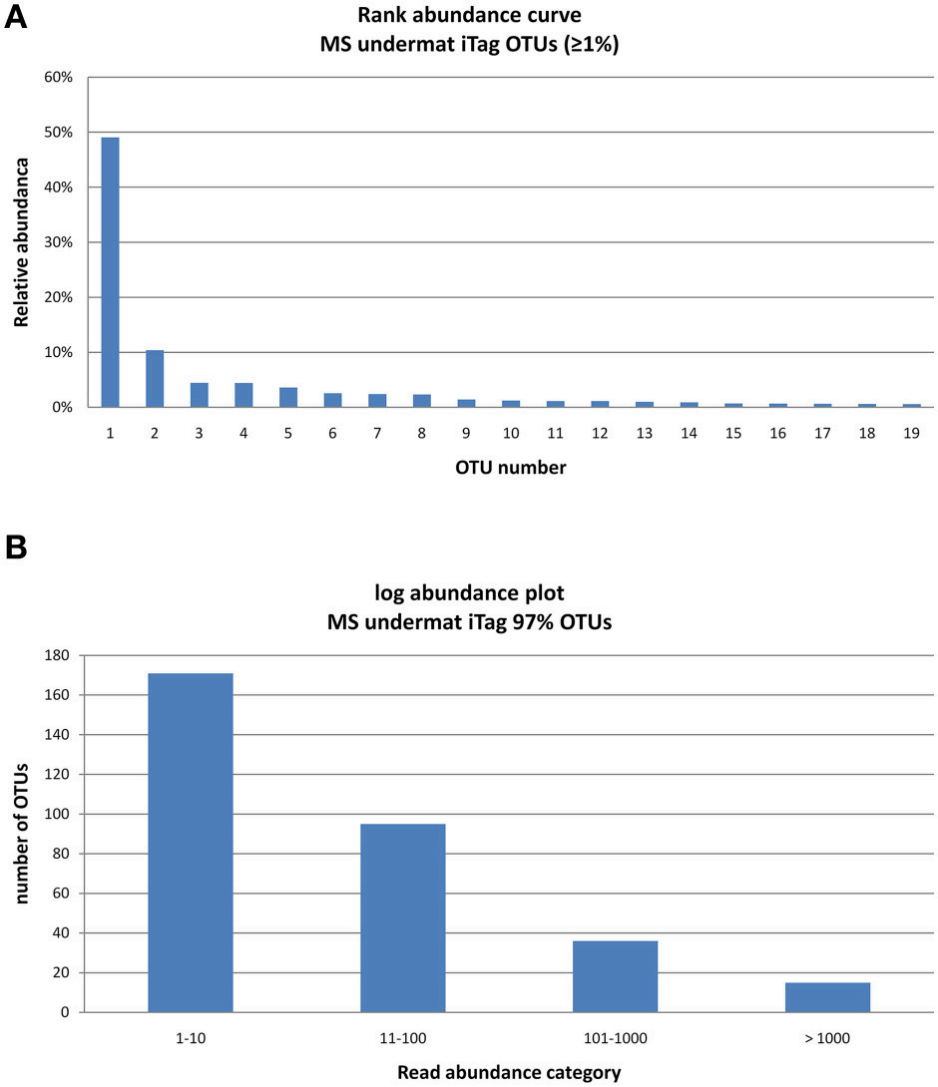
**Rank abundance curve of the 15 very abundant OTUs (>1,000 reads) obtained from the undermat 16S rRNA gene amplicon study (relative read counts) (A), and Log abundance plot of the all 317 OTUs obtained from the undermat 16S rRNA gene amplicon study (B)**. 15 OTUs (= 5%) were detected more than 1,000 times.

**Figure 4:**
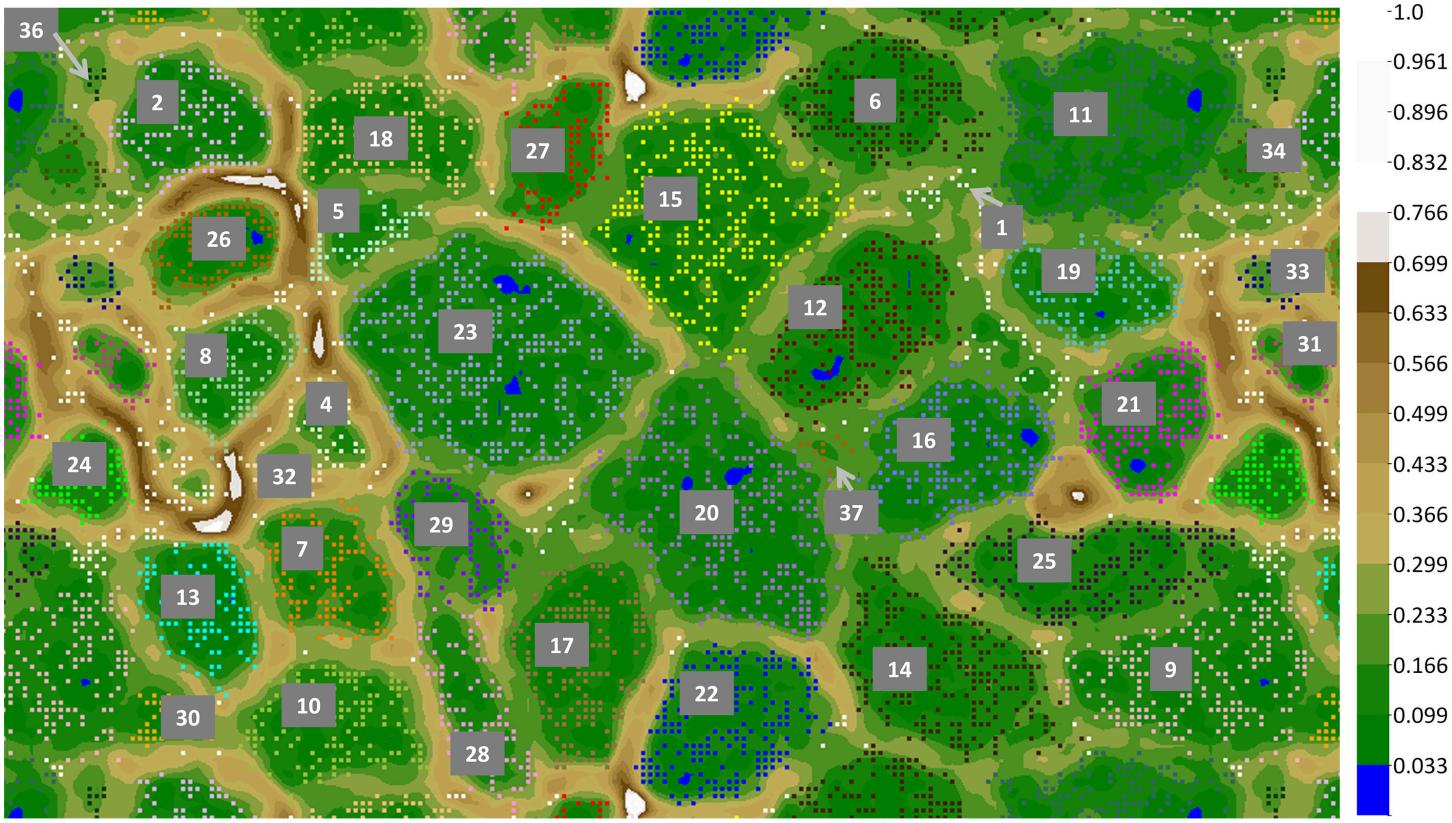
**ESOM binning of Mushroom Spring assembled undermat metagenome sequences >5 kb**. In order to show complete bins, the map is shown in tiled mode, displaying four connected partial copies of the grid with some redundancy of data points. Repeating colors imply repeating bins. Complete bins are labeled with numbers (number code and details of the bins is found in Table 3). Additional partial copies of the bins due to the tiled display are not labeled. The color gradient code on the right visualizes relative height values; the largest height normalized to 1.

**Figure 5:**
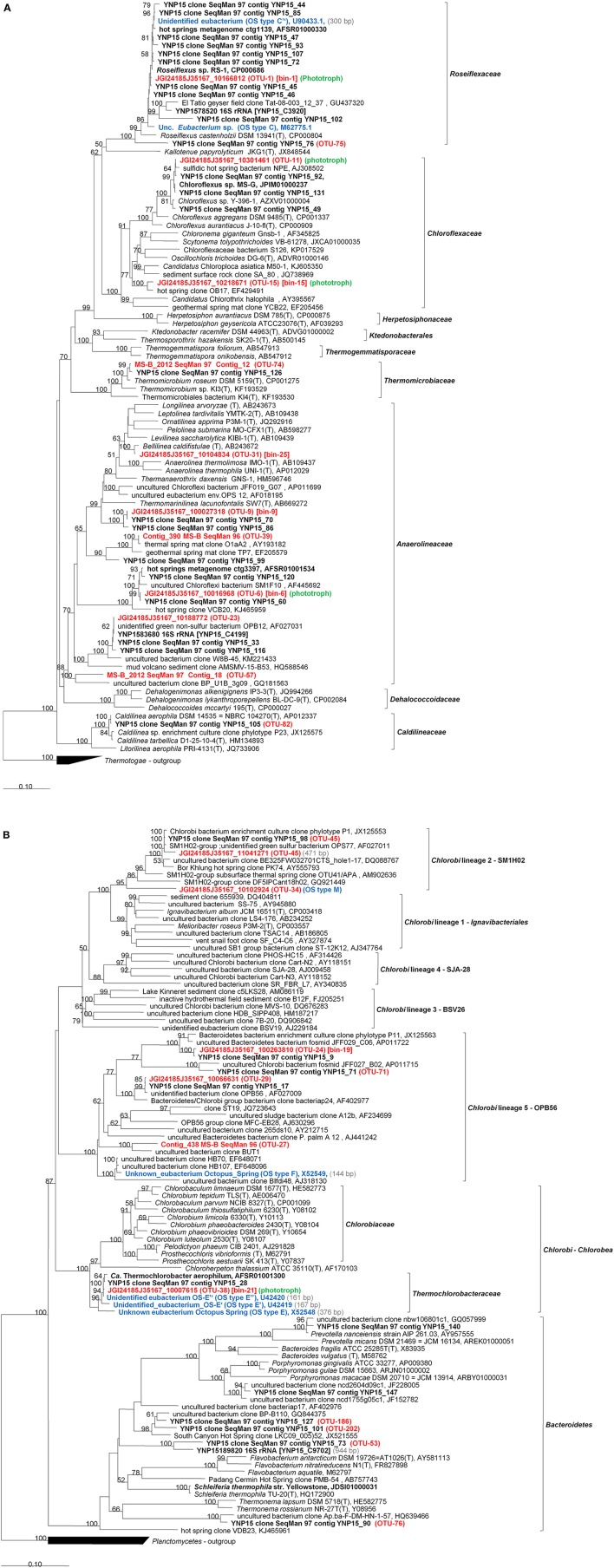
**Phylogenetic tree based on 16S rRNA gene sequences showing the phylogenetic relationship between members of the phylum Chloroflexi (A) and Bacteroidetes-Chlorobi (B) phyla and sequences obtained from the Mushroom Spring microbial undermat community**. The tree was generated based on the Maximum Likelihood method using the phyML software included in the ARB package. Percentage numbers on nodes refer to 100 bootstrap pseudoreplicates conducted. Only values >50% are shown. Bold sequences were obtained from Mushroom or Octopus Spring in this or previous studies. Red bold labels indicate sequences obtained in this study. Blue bold labels indicate “OS type” sequences from previous studies. OTU numbers shown refer to the most abundant OTU represented by the sequence. Only sequences with length >1,000 bp were used for phylogenetic calculations. Sequence length <1,000 bp are given in (gray) in the labels and corresponding sequences were added using the Parsimony method without changing tree topology.

We used deep sequencing of rRNA gene amplicons (iTags) and total environmental DNA to study the subsurface community of the chlorophototrophic microbial mat at Mushroom Spring. We describe the diversity and community composition on both levels, based on “OTUs” (Figures 2A, 3A, Table 1 and Table S1) and based on “dereplicated iTag” sequences (Figures 2B, 3B, Table 2) in Section “16S rRNA Gene Amplicons (iTags),” as well as on metagenomic bins obtained based on oligonucleotide frequency patterns in Section “Metagenome Sequencing” (Figure 4, Table 3). An overview of the most important taxa detected in each phylum will be presented in Section “Overview of Phyla and Taxa Detected in the Mushroom Spring Undermat.” Each iTag OTU was found to represent a variable number of dereplicated iTtag sequences, which is interpreted as representing different degrees of microdiversity within a taxon (Figure 2B, Table 1). Members of 20 different phyla were identified (Figure 5 and Figure S1, Table S1). Organisms of the phylum *Chloroflexi* dominated the microbial undermat community in both read abundance and diversity (Tables 1, 2, and Table S1, Figures 2A,B). Thirteen out of seventeen members of the microbial mat detected in previous 16S rRNA gene sequence cloning and DGGE studies (OS types, Table 4; Ward et al., 1990, 1992; Weller et al., 1992; Ferris et al., 1996b, 1997; Ferris and Ward, 1997), as well as relatives of ribosomal sequence types derived from a previous undermat study (Klatt et al., 2013a, Figure 5 and Figure S1) were detected in this study and thus confirmed as members of a compositionally and temporally stable microbial community.

**Table 1:** **Most abundant OTUs (97% nt identity), number of reads and relative abundance, microdiversity in terms of represented dereplicated iTag sequences, corresponding metagenome sequences and next relatives determined by BLAST search**.

**OTU-name**	**Reads**	**Rel. abundance (%)**	**No. of derep. iTags**		**Metagenome 16S rRNA**			**Relevant Blast hits**	**Acc. No**.	**Ident (%)**
			**Total**	**>100**	**Scaffold ID**	**Avg. coverage**	**Length (bp)**			
OTU-01	68,369	49	6,193	24	101,6681	2,842	1,357	*Roseiflexus* sp. RS-1	NR_074197	100
OTU-02	14,480	10	1,664	3	1,000,336	458	1,426	*Thermotoga hypogea* NBRC 106472 (T)	AP014508	98
OTU-03	6,203	4	2,082	9	1,062,246	951	710	Unc. bacterium clone SM2D03	AF445720	100
								*Eubacterium* sp. (OS type L)	L04707	98
OTU-04	6,160	4	899	3	1,002,657	262	1,434	clone YNP_SBC_BP2A_B2	HM448202	99
								*Thermocrinis ruber* strain DSM 23557(T)	CP007028	97
OTU-05	5,046	4	1,289	7	1,008,881	105	1,002	*Synechococcus* sp. JA-2-3B'a(2-13)	CP000240	100
OTU-06	3,580	3	675	1	1,001,696	423	1,364	clone YNP_SBC_BP4_B2	HM448255	98
								*Thermanaerothrix daxensis* GNS-1 (T)	HM596746	87
OTU-07	3,350	2	564	1	1,014,288	189	1,444	OP9 bacterium clone TP29	EF205555	98
								*Ammonifex thiophilus* strain SR(T)	EF554597	83
OTU-08	3,283	2	705	2	1,000,748	135	1,413	clone SMD-B01	AB477993	99
								*Thermodesulfovibrio yellowstonii* DSM11347(T)	NR_074345	96
OTU-09	1,981	1	449	2	1,000,273	129	1,389	clone NY-30	KC290430	94
								*Thermanaerothrix daxensis* GNS-1 (T)	HM596746	91
OTU-10	1,715	1	510	6	1,001,962	67	1,406	EM3 clone OPB88	AF027006	99
								*Rhodothermus marinus* SG0.5JP17-172 (T)	CP003029	82
OTU-11	1,594	1	521	3	1,030,146	83	1,309	*Chloroflexus* sp. MS-G	KR230107	99
								*Chloroflexus aurantiacus* J-10-fl (T)	CP000909	95
OTU-12	1,569	1	477	3	1,003,586	802	1,415	OP10 clone OPB80	AF027089	94
								*Fimbriimonas ginsengisoli* Gsoil 348 (T)	CP007139	81
OTU-13	1,392	1	306	2	1,015,572	36	1,497	clone bac67	HM184963	95
								*Thermoanaerobaculum aquaticum* MP-01 (T)	NR_109681	88
OTU-14	1,260	1	322	3	1,003,293	60	1,420	clone TP5	EF205581	99
								*Thermogutta terrifontis* R1 (T)	KC867694	90
OTU-15	1,008	1	391	3	1,021,867	132	1,299	clone OB17	EF429491	98
								*Chloroflexus aurantiacus* J-10-fl(T)	CP000909	90

**Table 2:** **Most abundant dereplicated iTag sequences (100% nucleotide identity) detected in the Mushroom Spring undermat**.

**MSunder_iTag (dereplicated iTag)**	**Reads**	**Relative abundance (%)**	**Phylum**	**Genus**	**OTU**
MSunder_iTag-1	30,285	21.70	*Chloroflexi*	*Roseiflexus*	1
MSunder_iTag-2	11,586	8.30	*Chloroflexi*	*Roseiflexus*	1
MSunder_iTag-3	8,257	5.90	*Thermotogae*	*Pseudothermotoga*	2
MSunder_iTag-4	4,712	3.40	*Chloroflexi*	*Roseiflexus*	1
MSunder_iTag-5	3,760	2.70	*Aquificae*	*Thermocrinis*	4
MSunder_iTag-6	2,551	1.80	*Chloroflexi*	*Ca*. Roseilinea	6
MSunder_iTag-7	2,436	1.70	*Atribacteria*		7
MSunder_iTag-8	2,229	1.60	*Armatimonadetes*		3
MSunder_iTag-9	1,881	1.40	*Chloroflexi*	*Roseiflexus*	1
MSunder_iTag-10	1,721	1.20	*Nitrospirae*	*Thermodesulfovibrio*	8
MSunder_iTag-11	1,716	1.20	*Cyanobacteria*	*Synechococcus*	5
MSunder_iTag-12	1,695	1.20	*Thermotogae*	*Pseudothermotoga*	2
MSunder_iTag-13	1,542	1.10	*Armatimonadetes*		3
MSunder_iTag-14	1,370	1.00	*Thermotogae*	*Pseudothermotoga*	2
MSunder_iTag-15	1,220	0.90	*Chloroflexi*	*Roseiflexus*	1
MSunder_iTag-16	1,161	0.80	*Chloroflexi*		9
MSunder_iTag-17	1,027	0.70	*Aquificae*	*Thermocrinis*	4

*Read numbers, relative abundance, taxonomic affiliation and OTU affiliation are provided*.

**Table 3:** **Metagenome bins recovered based on tetranucleotide frequencies**.

Bin^*^	**OTU**	**Identity**	**16S rRNA**	**Contigs**	**Size [Mb]**	**Marker genes**
1	OTU-01	*Roseiflexus* sp.	No	18	0.20	None
2	OTU-02	*Thermotoga* sp.	Yes, scaffold00336	142	1.85	30
3^$^	OTU-03	*Armatimonadetes* (OP10)	Yes	81	2.9	32
4	OTU-04	*Aquificae* / *Thermocrinis* sp.	Yes, scaffold02657	44	0.22	5
5	OTU-05	*Synechococcus* sp. B'	No	68	0.43	13
6	OTU-06	“*Ca*. Roseilinea gracile”	No	188	1.71	25
7	OTU-07	*Atribacteria* (OP9)	No	136	1.45	26
8	OTU-08	*Thermodesulfovibrio* sp.	Yes, scaffold00748	119	1.59	17
9	OTU-09	*Chloroflexi, Anerolineae*	Yes, scaffold00273	220	2.62	26
10	OTU-10	*Thermotogae*-EM3/OPS-2	Yes, scaffold01962	157	1.38	18
11	OTU-11	*Chloroflexus* sp. MS-G	No	336	3.1	21
12	OTU-12	*Armatimonadetes*_Gp6 (OP10)	Yes, scaffold03586	200	1.72	24
13	OTU-13	*Aminicenantes* (OP8)	No	94	2.54	30
14	OTU-14	*Planctomycetes*	Yes, scaffold03293	258	1.90	19
15	OTU-15	“*Ca*. Chloranaerofilum corporosum”	No	299	2.26	19
16	OTU-17	*Chloracidobacterium thermophilum*	No	220	2.07	21
17	OTU-18	*Armatimonadetes*_Gp 2	Yes, scaffold00584	207	2.64	31
18	OTU-21	*Meiothermus* sp.	No	155	1.31	8
19	OTU-24	*Chlorobi*-lineage 5: OPB56	Yes, scaffold02638	137	1.17	18
20	OTU-36	Acidobacterium, OPB3	Yes, scaffold01343	253	2.60	6
21	OTU-38	“*Ca*. Thermochlorobacter aerophilum”	No	198	2.09	27
22	OTU-46	*Elioraea* sp.	No	229	2.02	26
23		*Planctomycetes*	No	385	3.8	29
24		*Ignavibacteriaceae*	No	75	2.50	31
25	OTU-31	*Chloroflexi, Bellilinea sp*.	No	237	2.46	24
26		Unidentified	No	130	1.78	28
27		Unidentified	No	96	0.85	2
28		Unidentified	No	110	0.84	3
29		Unidentified	No	98	0.66	5
30		Unidentified	No	22	0.27	None
31		Unidentified	No	38	0.26	2
32	OTU-26	*Thermodesulfobacteria*	No	27	0.18	12
33		Unidentified	No	23	0.17	none
34		Unidentified	No	23	0.17	none
35		Unidentified	No	17	0.11	8
36		Unidentified	No	12	0.09	none
37		*Chloroflexi, Dehalocoocoides*-like	No	8	0.06	none

* *Numbers refer to ESOM bins shown in Figure 4*.

$ Bin 3 was obtained from an enrichment culture, not from the undermat metagenome.

**Table 4:** **OS-type sequences from previous studies (Ward et al., 1990, 1992; Weller et al., 1992; Ferris et al., 1996b, 1997; Ferris and Ward, 1997) and the corresponding sequences obtained in this study**.

**OS type**	**Acc. Nos**.	**Phylum**	**Identity**	**OTU**	**Metagenome sequences [JGI24185J35167_]**
A	X52544	*Cyanobacteria*	*Synechococcus* sp. A	OTU-22	10825551 (280 bp) 10154254 (272 bp) 10858251 (546 bp)
B	M62776 X52545	*Cyanobacteria*	*Synechococcus* sp. B'	OTU-5	12370211 (272 bp)
C	X52546 U42421 U90433	*Chloroflexi*	*Roseiflexus* sp. RS-1	OTU-1	10166812 (1357 bp)
D	X52547	*Acidobacteria*	*Chloracidobacterium thermophilum*	OTU-17	10032584 (1383 bp)
E	X52548 U42419 U42420	*Chlorobi*	*Ca*. Thermochlorobacter aerophilum	OTU-38	10007615 (1379 bp)
F	X52549	*Chlorobi*	*OPB56-like Chlorobi*	OTU-262	No scaffold
G	X52550	*Betaproteobacteria*	*Hydrogenophilus* sp.	OTU-101	No scaffold
H	X52551	*Sprichoaetes*	*Spirochaeta* sp.	OTU-41	No scaffold
I	L04709	*Cyanobacteria*	*Leptolyngbya* sp.	no OTU	No scaffold
J	L04710	*Cyanobacteria*	*Synechococcus* sp.	no OTU	1045912 (920 bp)
K	L04711	*Acidobacteria*	*Thermoanaerobaculum* sp.	OTU-61	10096124 (954 bp) 10037885 (587 bp)
L	L04707	*Armatimonadetes*	*Uncultured Armatimonadetes (OP10)*	OTU-3	11621781 (295 bp) 10622461 (710 bp) 12054061 (298 bp)
M	L04708	*Chlorobi*	Unidentified *Ignavibacteriaceae*	OTU-34	1068906 (652 bp) 1010292 (1038 bp)
N	L05931 L05930	*Betaproteobacteria*	*Tepidimonas* sp.	No OTU	No scaffold
O	L04706	*Alphaproteobacteria*	*Elioraea* sp.	OTU-46	10309321 (1157 bp) 10459122 (309 bp)
Q	U42422	*Chloroflexi*	*Candidatus* Chlorothrix halophila	No OTU	No scaffold
R	U46750	*Betaproteobacteria*	*Uncultured Beta-Proteobacterium*	OTU-172	No scaffold

### 16S rRNA gene amplicons (iTags)

Sequencing of partial 16S rRNA genes resulted in 139,326 total and 30,861 dereplicated (i.e., unique) reads after quality control. Abundance values of dereplicated reads varied between 1 and 30,285, with an average of 5.4 reads per sequence.

#### Diversity based on OTUs

The 16S rRNA gene amplicon reads clustered into 317 OTUs of ≥97% nt identity, with abundances between 1 and 68,369 reads per OTU (Table S1). The community was characterized by a low degree of evenness (Figure 3A). The majority of the OTUs were present in low abundance; only 15 OTUs (5% of the taxa) were represented by 1,000 or more reads (Figure 3B). Due to the high number of singleton sequences, the estimated richness based on Chao1 (S_chao1_ = S_obs_+ (no. of singletons^2^)/(2^*^ no. of doubletons) (Chao, 1984) was rather high, Chao1 = 369.74; a lower value of Chao1 = 220.9 was obtained in a previous study (Klatt et al., 2013a). In contrast, the Simpson's Reciprocal Index ($D=\frac{\sum n\left(n-1\right)}{N\left(N-1\right)}$) obtained in this study is considerably lower than in previous studies (3.85 in this study vs. 37.5; Klatt et al., 2013a), reflecting the low evenness and strong dominance of only a few OTUs in the amplicon study. While an identity cut-off of 97% for rRNA gene sequences is often used to demarcate species (Stackebrandt and Goebel, 1994; Schloss and Handelsman, 2005; Koeppel and Wu, 2013), this is an arbitrary value that does not necessarily correlate with any species definition. Here, we refer to OTUs as “taxa,” use the term “populations” mainly for dereplicated iTag sequences, and discuss our understanding of the bacterial species concept in Section “Discussion.”

#### Most abundant taxa based on OTUs

When considering OTU sequences based on 97% nt sequence identity, 15 OTUs were identified with >1,000 reads each, varying in abundance between 1,008 and 68,369 reads (Table 1). These are considered to represent highly abundant taxa and thus are likely to represent key members of the Mushroom Spring undermat community. However, the threshold of 1,000 reads was arbitrarily chosen and does not necessarily correlate with activity or ecological importance. We will focus the discussion on the “very abundant” taxa listed in Table 1, but will also include selected “abundant” and “less abundant” OTUs with read abundances of ≥100 and less, respectively (Table S1).

The 16S rRNA gene amplicons of the microbial undermat community were dominated by sequences derived from *Roseiflexus* spp. (Figure 2A, OTU-1, 49%) with the second most abundant sequences belonging to a *Pseudothermotoga* sp. (OTU-2, 10%). An unidentified *Armatimonadetes* (formerly known as OP10) bacterium (OTU-3), a member of the *Aquificae* (OTU-4), as well as the sequences derived from member of the *Cyanobacteria* each represented ~4% of the sequences (Table 1). On the basis of *psaA* sequences the cyanobacterial sequences can be classified as belonging to ecotype populations of *Synechococcus* detected in the upper green layer of the mat and are considered likely to arise from buried surface populations that are not expected to represent metabolically active constituents of the undermat community. The sixth most abundant OTU was identified as a phototrophic member of the phylum *Chloroflexi*, which had previously been detected in the upper green layer using metagenome analysis and identified as the first phototrophic “*Anaerolineae*-like” *Chloroflexi*; it has provisionally been named “*Ca*. Roseilinea gracile” (Klatt et al., 2011, 2013b; Tank et al., in press). Additional abundant OTUs were affiliated with the *Atribacteria* (OP9) *Nitrospirae, Planctomycetes* and several phototrophic and non-phototrophic members of the phylum *Chloroflexi* (Table 1). Three of the fifteen most abundant OTU sequences from the undermat amplicon study represented sequences obtained from the mats of Octopus Spring in previous 16S rRNA gene surveys (OS-B: *Synechococcus* sp. Type B; OS-C: *Roseiflexus* sp. RS-1; and OS-L: *Armatimonadetes* member OTU-3) (Table 4, Ward et al., 1990, 1992; Ferris et al., 1996a; van der Meer et al., 2010).

#### Most abundant populations based on dereplicated iTag sequences

Seventeen dereplicated iTag sequences, representing members of the nine most abundant OTUs, were each detected more than 1,000 times, and in total represent more than half of all iTag reads recovered in this study (Table 2, Figure 2B). These sequences probably correspond to the most abundant “populations” (in contrast to “taxa” for OTUs). Five of these very abundant dereplicated iTag sequences belong to a single OTU representing the most abundant taxon, *Roseiflexus* spp. (Figure 2B, Table 2 OTU-1, MSunder_iTags-1, 2, 4, 9, and 15). The third most abundant sequence (MSunder_iTag-3), as well as two additional abundant, dereplicated sequences (MSunder_iTags-12 and 14), were representatives of *Pseudothermotoga* spp. (OTU-2), the second most abundant taxon. The *Armatimonadetes* (OTU-3) and a member of the phylum *Aquificae* (OTU-4) contained two slightly different, highly abundant dereplicated iTag sequences each, whereas the other OTUs (OTUs 5–9) had only one very abundant dereplicated iTag sequence. With regard to the single dereplicated iTag sequences, cyanobacteria derived from the green upper layer of the mat community are represented by the eleventh most abundant iTag sequence, and thus the ten most abundant dereplicated iTag sequences (representing eight OTUs) are considered to represent the most abundant populations in the undermat community (MSunder_iTag-1 through MSunder_iTag-10; Table 2).

#### Microdiversity

We used different methods to assess the degree of sequence heterogeneity and microdiversity within the microbial undermat community. Based on the number of different dereplicated iTag sequences within one 97% OTU, a high degree of diversity was indicated, especially for the most abundant OTU, *Roseiflexus* spp. We detected 6,193 total dereplicated iTag sequences, 24 of which had >100 reads (Table 1). A similar microdiversity was identified by the oligotyping approach, and was also suggested by a high number of very similar but non-identical clone sequences obtained in a previous study (Klatt et al., 2013a; Figure 5A, and Figure S2, Table S2). Based on ten distinct nucleotide positions, 246 different oligotypes were identified, of which 55 were represented by >10 reads, 23 by >100 reads and nine by > 1,000 reads in the combined dataset (which consisted of ~39,000 upper green layer reads and 75,000 undermat reads). The total “purity scores” of 0.95 and 0.86 for >100 and >10 reads, respectively, indicates a good separation for the highly abundant oligotypes, but also implies further low abundance oligotypes in the samples. Differences in diversity and abundance of oligotypes between the upper green layer and the undermat were detected, e.g., for the most abundant *Roseiflexus*-oligotypes (Table S2, Figure S2). In general the undermat is more diverse. The upper green layer for example contains a lower number of highly abundant oligotypes (six oligotypes >1% of all *Roseiflexus* sequences), whereas the undermat is more diverse with nine oligotypes >1% (Table S2, Figure S2). Notably, the most abundant oligotypes are present in both samples in similar abundances. One oligotype dominates both datasets (48% in the upper layer vs. 54% in the undermat). The second most abundant oligotype “CTCTACGGGC” is more abundant in the upper layer (32 vs. 20% of the reads), whereas the third is more abundant in the undermat (9 vs. 6%, Table S2). In general the undermat is more diverse and some oligotypes show distinct differences. For example, the difference in the entropy figures from upper green layer and undermat after separate analyses (considerably lower entropy at pos. 104 and 109 in upper layer; Figures S2B,C) are indicative of a lower abundance of two oligotypes in the upper layer, namely “CCCCGCGTGC” (2.13% in undermat, 0.19% in upper layer) and “CCCCGCGGGC” (1.02 vs. 0.21%) (Table S2).

A high degree of microdiversity was also indicated for other OTUs obtained in this study, e.g., OTU-3 (*Armatimonadetes* member, OS type L) and OTU-5 (*Synechococcus* spp.) Overall, the twelve most abundant OTUs also exhibited the highest number of unique amplicon sequences, indicating a correlation between microdiversity and sequencing depth (Table 1). However, the number of abundant dereplicated sequences, i.e., putative ecotypes did not show the self-correlation with sequencing depth, but correlated with the metagenome assembly quality; a high microdiversity was suggested to be interfering with the sequence assembly. Very few contigs with >5 kb length were assembled for the OTUs with the highest microdiversity (OTU-1 and OTU-3).

### Metagenome sequencing

One full lane of Illumina HiSeq sequencing led to 176,741,874 quality-passed reads. 169,595,919 (96%) of these reads were assembled into a 232-Mb metagenome comprising 315,154 total contigs with a maximum scaffold length of 158 kb and a N/L50 value of 32,529/1.24 kb, which defines the number of fragments at or above the Length50 cutoff. There were 13,766 contigs >2.5 kb, 5,362 contigs >5 kb, and 1,665 >10 kb. Contigs >50 kb (*n* = 38) accounted for 1.14% of all assembled sequence data.

#### Metagenome bins

Binning of the metagenome contigs based on tetranucleotide frequency patterns resulted in 36 clusters (Table 3, Figure 4). An additional bin, representing OTU-3 from the iTag study of the undermat, was obtained from an cyanobacterial enrichment culture metagenome (Olsen et al., 2015). Thus, 37 partial genomes, 26 of which contained ≥1 Mb of sequence information, were found by this method (Table 3). Twenty-six of the bins were identified taxonomically, and 22 could be affiliated with abundant OTUs. A specific cut-off with regard to taxonomic levels or sequence threshold cannot be given for the represented populations. However, previous studies, as well as joint binning of the sequences from the presented study with reference genomes, suggest that genomes derived from bacterial populations with 16S rRNA gene sequences identities of ≥96% do not separate into distinct bins (data not shown; Klatt et al., 2011). In this study, the cyanobacterial genomes of *Synechococcus* Types A and B' (97% 16S rRNA nt identity), and within the *Chloroflexi, Roseiflexus castenholzii* and *Roseiflexus* sp. RS-1 (95.6% 16S rRNA nt identity) as well as *Chloroflexus aurantiacus* J-10-fl and *Chloroflexus* sp. MS-G (95.7% 16S rRNA nt identity) genomes clustered in single bins containing sequences of both genomes, respectively. All other included *Chloroflexi* reference genomes (<94% 16S rRNA nt identity) clustered in separate but sometimes adjacent bins. The occurrence of several metagenomic bins affiliated with the *Chloroflexi* as well as the separate clustering of the included *Chloroflexi* reference genomes, provides an estimate of the ability of this approach to discriminate and resolve among different members of the same phylum. Based on these observations, as well as 16S rRNA OTU similarities found in this study displaying values of either <95% or >96.8% nt identity, we expect genomes of populations sharing <95% 16S rRNA sequence identity to be represented by distinct metagenomic bins, whereas OTUs of >96.8% similarity would probably be represented by a single partial genome (i.e., metagenomic bin).

### Overview of phyla and taxa detected in the mushroom spring undermat

In the following paragraphs we will describe selected taxa from each phylum detected in the undermat community based on combined information of iTag and metagenomic sequence data. The phyla and members thereof are presented in the order of abundance, starting with the most abundant phylum and the most abundant member, respectively. Taxonomic identification was always based on the longest 16S rRNA sequence available, in conjunction with phylogenetic marker genes. Information on additional taxa and phyla can be found in the phylogenetic trees and the Supplemental Materials (Figure 5, and Figure S1, Table S1). Phylogenetic analyses based on 16S rRNA sequences extracted from metagenomic data identified >50 members of 20 different phyla (Figure 5 and Figure S1), most of which could also be affiliated with iTag sequences obtained in the amplicon study.

#### Chloroflexi

Members of the phylum *Chloroflexi* were the most diverse group of organisms present in the microbial undermat community. Overall, 41 OTUs were affiliated with the phylum *Chloroflexi* (Table S1), and twelve *Chloroflexi* sequences were identified phylogenetically (Figure 5A). Five of the fifteen most abundant OTUs (>1,000 reads), as well as four abundant OTUs with ≥100 reads, were identified as members of the *Chloroflexi* (Table S1, Figure 5A). Based on the metagenomic information for these taxa, four out of five very abundant *Chloroflexi* are chlorophototrophic members of this phylum (OTUs-1, 6, 11, and 15; see Figure 5A), while one is a putative chemoheterotroph (OTU-9). Three additional abundant OTUs also are associated with putatively chemoheterotrophic members of this phylum (OTUs 23, 31, and 39). Thirty-two less abundant OTUs were also affiliated with the phylum *Chloroflexi* (Table S1, Figure 5A).

Binning of the assembled metagenomic data yielded only a very small partial genome for *Roseiflexus* spp., the most abundant and most diverse OTU in the undermat (Bin-1; Figures 2, 3, 5, Tables 1–3 and Table S1). Bin-1 did not contain any phylogenetic marker genes but was identified by high nucleotide sequence identities (92 ± 5%; range 79–100%) to the *Roseiflexus* sp. RS-1 genome (CP000686, 5.8 Mb, van der Meer et al., 2010). The *Roseiflexus* sp. RS-1 genome recruited 23,534 contigs from the metagenome (≥85% nt identity and ≥75 coverage), of which 13,329 contigs showed sequence identity of ≥95%. Only 12 of those contigs were >5 kb in length, sharing a minimum of 94.52% nt identity with the *Roseiflexus* sp. RS-1 genome sequence. *Roseiflexus* sp. RS-1 is a filamentous anoxygenic phototroph that synthesizes bacteriochlorophyll (BChl) *a* but not BChl *c*. It was previously isolated from Mushroom Spring and was affiliated with OS Type C sequences obtained in early molecular studies (Ward et al., 1990; Ferris et al., 1996b, 1997; Ferris and Ward, 1997). In addition to BChl *a*-containing photosynthetic reaction centers, the genome of this organism encodes xanthorhodopsin, which was also detected in the undermat metagenome (RoseRS_2966, GenBank Acc. no. ABQ91330.1; JGI24185J3567_10248071), and indicates a possible additional use of light energy (Choi et al., 2014). The small number of long contigs affiliated with this OTU, in combination with the broad coverage range from 31 × to 1,557 ×, reflects a high microdiversity as well as the high abundance of the core genome sequences.

A 1,364-bp partial 16S rRNA sequence identified OTU-6 as a member of the *Chloroflexi*, which is most closely related to uncultured members in streamer biofilm-producing communities in YNP hot springs (Table 3; Meyer-Dombard et al., 2011). It represents an uncultured chlorophototrophic *Anaerolineae*-like organism, which was also identified in the upper green layer of the Mushroom Spring microbial mat in a previous metagenomic analysis (Klatt et al., 2011). Despite the absence of a 16S rRNA gene, Bin-6 was identified to represent OTU-6 based on 93 ± 5.6% average nt identity to Cluster 6 from the upper layer metagenome (Klatt et al., 2011), which did contain a ribosomal RNA sequence with 98% identity to OTU-6, as well as 99% sequence identity to a 16S rRNA sequence detected in the metagenome of this study. When first reported by Klatt et al. (2011), this uncultured organism was identified as “*Anaerolineae*-like,” with *Anaerolinea thermophila* strain UNI-1 being its closest cultivated and described relative (85% nt identity, Sekiguchi et al., 2003). At the time of this writing [February 2016], a BLAST search identified *Thermanaerothrix daxensis* strain GNS-1^T^ (Grégoire et al., 2011) and *Thermomarinilinea lacunofontalis* strain SW7 (Nunoura et al., 2013) as the closest isolated relatives with a 16S rRNA sequence identity value of 87% (Table 1). Phylogenetic analysis based on the full-length 16S rRNA sequences supports a phylogenetic affiliation to the *Anaerolineales* as well as a more distant relationship to known chlorophototrophic *Chloroflexi* (Figure 5A). Genes annotated within this metagenomic bin suggest that, like *Roseiflexus* spp., this anoxygenic chlorophototroph has the potential to produce BChl *a* but probably doesn't contain BChl *c* or chlorosomes, although it does possess a putative xanthorhodopsin-like gene (Klatt et al., 2011). Thin short filaments possibly representing this *Anaerolineae*-like phototrophic *Chloroflexi*, tentatively named “*Ca*. Roseilinea gracile” (Tank et al., in press), have been observed in fresh mat samples and enrichment cultures. They exhibit BChl *a* but not BChl *c* autofluorescence.

OTU-09 is represented by Bin-9 and was also identified as being derived from a member of a cluster of uncultured *Chloroflexi* within the *Anaerolineae* (Figure 5A). However, based on the absence of photosynthesis-related genes in the corresponding metagenomic bin and the absence of unassigned photosynthesis-related genes in the remaining unbinned contigs, the organisms corresponding to OTU-09 are not predicted to be chlorophototrophs.

A close relative of *Chloroflexus* sp. strain MS-G, a chlorophototrophic member of the *Chloroflexi* that was previously isolated from this mat (Thiel et al., 2014b), is represented by OTU-11 and Bin-11 in this study. Like strain MS-G, OTU-11 is predicted to be an anoxygenic phototroph containing type-2 (quinone-type) photosynthetic reaction centers, light-harvesting complex 1 and chlorosomes based on a metagenomic bin of 3.1 Mb, with an average read coverage of 30 × (Bin-11, Table 3). The bin contained 21 phylogenetic marker genes, all of which share amino acid sequence identity values of 98.7 to 100% with sequences from *Chloroflexus* sp. MS-G (Table 3). The organism representing OTU-11/Bin-11 and strain MS-G share 98.3% 16S rRNA and 94 ± 6% overall genomic nucleotide sequence identity, respectively.

A third anoxygenic phototrophic *Chloroflexi* is represented by OTU-15 and Bin-15. Phylogenetic analysis and BLAST search results indicate this organism to be only distantly related to other chlorophototrophic *Chloroflexus* spp., displaying 90–91% 16S rRNA sequence identity to *Oscillochloris trichoides, Chloroflexus aurantiacus* J-10-fl and “*Candidatus* Chloroploca asiatica.” The organism associated with these sequences presumably represents a novel genus of chlorophototrophic *Chloroflexi* within the family *Chloroflexaceae* (Figure 5A). Based on the conserved signature indels that are specific for different groups within the *Chloroflexi* as described by Gupta et al. (2013), this filamentous anoxygenic phototroph is affiliated with the proposed order of “green nonsulfur bacteria,” *Chloroflexales*, suborder *Chloroflexineae*, but is distinct from all known members of the genera *Chloroflexus* and *Oscillochloris*. The functional gene content of the associated metagenome bin (Bin-15) indicates that this organism has the capacity to synthesize BChls *a* and *c*. A filamentous BChl *a*- and BChl *c*-producing isolate similar to *Oscillochloris* sp. has been obtained in enrichment cultures, and tentatively named “*Candidatus* Chloranaerofilum corporosum” (Tank et al., in press).

*Thermomicrobium roseum*, phylum *Chloroflexi*, which had previously been isolated from the mats (Jackson et al., 1973), was detected in the metagenome in this study and a previous 16S rRNA cloning study (Klatt et al., 2013a), but *T. roseum* was only present in low numbers based on the analysis of iTag amplicons (OTU-74, 44 reads, Table S1, Figure 5A).

#### Thermotogae

Only two OTUs, OTU-2, and OTU-107, were identified as members of the phylum *Thermotogae* by the RDP classifier (Table S1). OTU-2 represents the second most abundant species-level iTag sequence and the corresponding metagenomic 16S rRNA sequence is 99% identical to that of *Pseudothermotoga hypogea*, formerly known as *Thermotoga hypogea* (Fardeau et al., 1997; Bhandari and Gupta, 2014). Bin-2 sequences, which represent this *Pseudothermotoga* sp. OTU-2 mat member (Table 3), show high similarities (98–100% aa sequence identities) to sequences obtained from a previous metagenomic study by Klatt et al. (2013a; IMG/M OID 2015219002), and form a single cluster with the genome sequence of *Pseudothermotoga hypogea* DSM 11164 in the metagenome binning analysis, which indicates the high similarity of these two genomes. OTU-107 shares 99% nt sequence identity to *Fervidobacterium pennivorans* strain DSM 9078 as well as to *Fervidobacterium* sp. isolated from YNP (Sullivan et al., unpublished, AY151268) but is represented by only 20 reads (Table S1, Figure S1). In addition, several sequences were affiliated with group EM3, which has tentatively been placed in the *Thermotogae* (Reysenbach et al., 2000) (Table S1, Figure S1). OTU-10 was misidentified as a member of the *Chlorobi* by RDP classifier, but actually represents the most abundant EM3 population and shares highest similarities with hot spring clones OPB88 (AF027006, Hugenholtz et al., 1998b) and OPS2 (AF018187, Graber et al., unpublished) from YNP with 99 and 98% 16S rRNA nt identity, respectively. Bin-10 representing this OTU was identified based on the presence of a matching 16S rRNA gene (Table 3). Phylogenetic affiliations of the phylogenetic marker genes were uncertain with most of the sequences only being assigned to the kingdom (“bacteria”) and phylum level (“*Bacteroidetes*,” “*Chlorobi*,” “*Deinococcus-Thermus*,” “*Chloroflexi*,” or “*Thermotogae*,” respectively), which indicates a high degree of novelty for this uncultured organism. Sequences similar to the ones in this metagenomic bin have previously been detected in the oxic upper green layer of the mat community (Klatt et al., 2011). The sequences formed unidentified Cluster 8 in the previous study, which were associated with an uncultivated, putatively heterotrophic bacterium. Bin-10 and Cluster 8 sequences formed a single bin when included in the analysis. A BLASTn comparison revealed an average nucleotide identity of 97 ± 3% between sequences of the previous cluster and the sequences in the bin from this study.

#### Armatimonadetes (OP10)

Uncultivated members of the Candidate phylum OP10, now named *Armatimonadetes* (Tamaki et al., 2011; Lee et al., 2013), were first detected in Obsidian Pool in YNP (Hugenholtz et al., 1998b). The undermat community at Mushroom Spring also contains a considerable diversity of members of this phylum. Two of the most highly abundant OTUs, OTUs 3, and 12, were identified as members of the *Armatimonadetes*. In addition, two abundant (OTUs 18 and 33) and nine less abundant iTag OTUs were identified as members of this phylum (Table S1). Partial genomes were identified for OTUs-3, 12, and 18 (Table 3, Figure S1).

Despite the high abundance of *Armatimonadetes* member OTU-3 sequences in the amplicon study and the presence of a partial 16S rRNA sequence with high coverage (951 ×; JGI24185J35167_1062246), no corresponding bin was obtained in the undermat metagenome. Serendipitously, a highly similar organism (99% 16S rRNA sequence identity) was identified as a chemoheterotrophic contaminant in a cyanobacterial enrichment culture obtained from these mats in the Ward laboratory at Montana State University (unpublished data). A partial genome of this enrichment contaminant was obtained by binning the assembled contigs of the corresponding enrichment culture metagenome (Bin-3, Table 3). This enrichment partial genome recruited 17,252 sequences (a total of 11 Mb of sequence data) from the undermat metagenome displaying 90.5 ± 7.5% nt id (covering min. 80% of the metagenome scaffold). OTU-3 amplicon sequences were also detected in the upper green layer in lower numbers (4.5 vs. 0.8% relative abundance; Table S1) and a partial genome of this organism was also detected as an unidentified heterotroph Cluster 7 in the upper layer metagenome (Klatt et al., 2011). The partial genome of the upper layer displayed similar identity values of 90.3 ± 7.5% to the enrichment culture metagenome bin and 94.6 ± 5.3% to sequences in the undermat metagenome, and formed a single ESOM bin with the partial genome obtained from the enrichment culture (data not shown). OTU-3 was phylogenetically identified as belonging to the “OS-L clade” within the uncharacterized group 7 of the phylum *Armatimonadetes* (Lee et al., 2013) (Figure S1). Clade OS-L is named after the first sequence of this clade, OS Type L, obtained from a DGGE study of enrichment cultures from microbial mats in Octopus Spring (Ward et al., 1992), with which the 16S rRNA genes in both Bin-3 from the enrichment culture and the undermat metagenome share 98% nt identity (L04707). So far, no isolated representative has been reported for this phylogenetic group. The presence of all 31 bacterial phylogenetic marker genes in the bin suggests that it contains a nearly complete genome (Table 3). Genes encoded in the partial genome, in combination with its occurrence in an enrichment with oxygenic cyanobacteria, indicates that this organism probably exhibits an aerobic or microaerobic lifestyle, similar to the other isolated members of the *Armatimonadetes* (Lee et al., 2011; Tamaki et al., 2011; Im et al., 2012). A considerable microdiversity was suggested by the presence of nine abundant iTag sequences (Table 1) as well as the diversity of partial, flagellum-associated genes affiliated with this organism, which were present on short contigs in the metagenome. Additionally, thirteen closely related 16S rRNA sequences were derived from a previous undermat 16S rRNA cloning study (Klatt et al., 2013a). These sequences show high identity values (>97%) to the OTU-3 sequence as well as to each other (assembly based on 97% nt sequence identity, Figure S1A) and also reflect a high microdiversity of these organisms. Similar to the situation found for *Roseiflexus* spp. (see above), the high microdiversity suggested for this taxon probably caused assembly difficulties, which may explain why no metagenomic bin was recovered directly from the undermat metagenome.

#### Aquificae

Of four OTUs identified as belonging to members of the *Aquificae* (Table S1), only OTU-4 was detected in significant numbers (Table S1). The corresponding 1,434-bp rRNA metagenomic sequence is 99% nt identical to clone sequences previously obtained from YNP hot spring habitats (*Thermocrinis* sp. clone YNP_SBC_BP2A_B2, HM448202, Meyer-Dombard et al., 2011), as well as to the YNP isolate *Thermocrinis* sp. P2L2B (AJ320219, Eder and Huber, 2002). The closest described relative is *Thermocrinis ruber* DSM 23557, which was isolated from Octopus Spring and which has a 16S rRNA sequence that shares 97% nt identity to the one found in this study (Huber et al., 1998) (Figure S1). Correlating to the high microdiversity detected for this OTU (Table 1), only a small partial genome was identified in the binning analysis of the metagenome (Bin-4, Table 3). The presence of at least two closely related populations in the undermat community is indicated by two highly similar (96% amino acid identity), *Thermocrinis*-like *soxB* genes; these genes are located on three individual scaffolds in the metagenome, each [gene-1, ~270 × coverage: JGI24185J35167_10446912, JGI24185J35167_10438521, JGI24185J35167_10819822; gene-2, ~70 × coverage: JGI24 185J35167_10446972, JGI24185J35167_10438611, JGI24185J35167_10820392], which also suggests problems with sequence assembly that could be related to microdiversity.

#### Cyanobacteria

The two major photoautotrophic primary producers of the upper green layer, *Synechococcus* spp. Type A and Type B', were also abundant members of the undermat by iTag analysis (OTUs 5 and 22, Table 1, Table S1). Seventeen additional but less abundant iTag OTUs (each ≤ 25 reads, representing <0.05% of the total iTag sequences) were assigned to cyanobacteria (Table S1). At the temperature sampled in this study (60°C), members of *Synechococcus* sp. Type B' (OS Type B', Table 4) are the predominant organisms (Klatt et al., 2011; Liu et al., 2011) and were also detected in this study (OTU-5, Bin-5, Table 3). *Synechococcus* sp. A (OS Type A, Table 4) sequences were detected in lower abundance (OTU-22, Table S1). The small size of Bin-5 (Table 3) reflects a low number of long and well-assembled contigs (68 contigs, 5,005–12,792 bp; 18 × to 96 × coverage) in comparison to a total of 3,353 contigs identified as having their origins in members of the *Cyanobacteria* in the metagenome (440 to 12,792 bp). Local BLASTn analysis and reference guided assembly using the genome sequence of *Synechococcus* sp. Type B' as query (applying a 95% nt identity threshold) identified 4,898 contigs as belonging to these organisms. The low assembly quality is indicative of high microdiversity as indicated by the presence of seven abundant iTag sequences (Table 1). Recent studies have found that a high number of ecotype populations occur within this cyanobacterial population, displaying variations in gene content and sequence as well as differences in gene arrangement (Becraft et al., 2011; Olsen et al., 2015). Genome sequences of several ecotypes isolated from the dominant cyanobacteria from Mushroom Spring are now available, and these provide comprehensive insights into the physiological and metabolic capacities of the oxygenic chlorophototrophs in the mat (Bhaya et al., 2007; Nowack et al., 2015; Olsen et al., 2015).

#### Atribacteria (OP-9/JS1)

The phylum *Atribacteria*, formerly known as Candidate phylum OP-9/JS1, exhibited low diversity. Of two OTUs identified as belonging to members of this phylum, only OTU-7 was detected in significant numbers in the iTag analysis (Table S1, Figure S1). OTU-7 represented 2.4% of all iTag reads and was represented by only a single abundant dereplicated iTag sequence (Table 1). Bin-7 contained a partial genome of this uncultured bacterium, as identified by the full-length 16S rRNA sequence which shared 99% and 98% sequence identity to *Atribacteria* clones OPB72 and TP29 obtained from hot springs in YNP and Tibet, respectively (Hugenholtz et al., 1998b; Lau et al., 2009). The affiliated metagenomic bin indicates an anaerobic, fermentative lifestyle for this member of the *Atribacteria* (data not shown), which is similar to properties deduced from single-cell genome sequences previously obtained from members of the *Atribacteria* (Dodsworth et al., 2013; Nobu et al., 2016).

#### Nitrospirae

iTag analysis identified seven *Nitrospirae* OTUs in the undermat community, of which only one, OTU-8, was abundant (Table S1). Bin-8 was assigned to this *Thermodesulfovibrio* sp.-like mat member based on presence of the corresponding 16S rRNA sequence (Figure 4, Table 3). OTU-8 represented ~2.0% (3,283 reads) of all iTag sequences (Table 1), and the full 16S rRNA sequence was most closely related to a clone sequence obtained from geothermal groundwater (99%, clone: SMD-B01, NCBI acc. no. AB477993, Kimura et al., 2010) and to *Thermodesulfovibrio yellowstonii* strain DSM 11347, as the closest isolated relative (96%, NCBI acc. no. CP001147, Henry et al., 1994; Bhatnagar et al., 2015). Bin-8 contained scaffolds with coverage values ranging from 29 to 135, which possibly reflects two different populations with different abundances. This was also suggested by the different read numbers of two abundant, dereplicated iTag sequences (OTU-8, iTag-10, 1,721 reads; and iTag-28, 602 reads; Table S1). The partial genome suggests sulfate-reducing metabolism for this organism, similar to *T. yellowstoneii*, which was isolated from thermal vent water in Yellowstone Lake, Wyoming, USA (Henry et al., 1994; Bhatnagar et al., 2015). The *dsrAB* gene sequences associated with dissimilatory sulfate-reduction of this uncultured organism have previously been detected in the Mushroom Spring microbial mat, and the corresponding *Thermodesulfovibrio*-like organism was associated with the sulfate reduction activity measured in the mat (Dillon et al., 2007). OTU-8 has been detected in both the upper and lower parts of the mat (Table 5, Table S1), possibly indicating that these organisms are not restricted to the undermat; this is further supported by the finding of *Thermodesulfovibrio*-like sequences also in the green upper layer metagenome in a previous study (Klatt et al., 2011).

**Table 5:** **Overview of community composition detected by the different methods used in this study (iTag, metagenome 16S rRNA, metagenome binning) and relative abundances in undermat and upper layer iTag sequencing study**.

**iTag OTU**	**Identity**	**Undermat metagenome 16S rRNA**	**Undermat metagenomic bin**	**iTag MS undermat (%)**	**iTag MS upper green layer (%)**	**Upper green layer metagenomic bin^a^**
OTU-01	*Chloroflexi, Roseiflexus* sp., OS Type C	Yes, scaffold 166812	Yes, bin-1 [very small]	49.1	33.9	Yes
OTU-02	*Thermotogae, Pseudothermotoga* sp.	Yes, scaffold 336	Yes, bin-2	10.4	1.5	No
OTU-03	*OP10/Armatimonadetes*_Gp7, OS Type L	Yes, scaffold 622461	No [enrichment bin-3]	4.5	0.8	Yes
OTU-04	*Aquificae, Thermocrinis sp*.	Yes, scaffold 2657	Yes, bin-4	4.4	0.3	No
OTU-05	*Cyanobacteria, Synechococcus* sp. B', OS Type B	Yes, scaffold 8881	Yes, bin-5	3.6	37.4	Yes
OTU-06	*Chloroflexi, Anaerolineae*-like' phototroph	Yes, scaffold 1696	Yes, bin-6	2.6	1.2	Yes
OTU-07	*OP9/Atribacteria*	Yes, scaffold 14288	Yes, bin-7	2.4	0.7	No
OTU-08	*Nitrospirae, Thermodesulfovibrio* sp.	Yes, scaffold 00748	Yes, bin-8	2.4	2.7	(Yes^b^)
OTU-09	*Chloroflexi, Anerolineae*	Yes, scaffold 273	Yes, bin-9	1.4	0.7	No
OTU-10	*Thermotogae*, EM3/OPS-2	Yes, scaffold 1962	Yes, bin-10	1.2	3.1	Yes
OTU-11	*Chloroflexus* sp. MS-G	Yes, scaffold 301461	Yes, bin-11	1.1	1.2	Yes
OTU-12	OP10/*Armatimonadetes*_Gp6	Yes, scaffold 3586	Yes, bin-12	1.1	0.5	No
OTU-13	OP-8/*Aminicenantes*	Yes, scaffold 32931	Yes, bin-13	1.0	0.0	No
OTU-14	*Planctomycetes*	Yes, scaffold 3293	Yes, bin-14	0.9	0.0	No
OTU-15	*Chloroflexi*	Yes, scaffold 218671	Yes, bin-15	0.7	0.0	No
OTU-16	*Planctomycetes, Gemmata*	Yes, scaffold 261011	Maybe, bin-23	0.7	0.2	No
OTU-17	*Chloracidobacterium thermophilum*	Yes, scaffold 32584	Yes, bin-16	0.7	5.2	No
OTU-18	*Armatimonadete*s_Gp 2	Yes, scaffold 584	Yes, bin-17	0.6	0.0	No
OTU-19	*Planctomycetes*, uncultured	Yes, scaffold 11289	No	0.6	0.0	No
OTU-20	*Elusimicrobia*, uncultured	Yes, scaffold 20130	No	0.5	0.1	Maybe
OTU-21	*Deinococcus-Thermus, Meiothermus* sp.	Yes, scaffolds 8341 and 25957	Yes, bin-18	0.5	0.4	No
OTU-22	*Cyanobacteria, Synechococcus sp. A*, OS Type A	Yes, scaffold 85825	No	0.5	0.5	No
OTU-23	*Chloroflexi, Anaerolineae, OPB12*	Yes, scaffold 18877	No	0.5	0.0	No
OTU-24	*Chlorobi, Lineage 5: OPB56*	Yes, scaffold 2638	Yes, bin-19	0.5	2.0	No
OTU-25	*Spirochaetae, Leptospiraceae*	Yes, scaffold 4665	No	0.4	0.1	No
OTU-26	*Thermodesulfobacteria, Caldimicrobium*	Yes, scaffold 193547	Yes, bin-32	0.4	0.4	No
OTU-27	*Chlorobi, Lineage 5: OPB56*	Yes, scaffold 104947	No	0.4	1.0	No
OTU-28	*Thermotogae, EM3*	Yes, scaffold 49539	No	0.4	0.0	No
OTU-29	*Chlorobi, Lineage 5: OPB56*	Yes, scaffold 6663	No	0.4	0.3	No
OTU-30	*Thermotogae, EM3*	Yes, scaffold 26263	No	0.4	0.0	No
OTU-31	*Chloroflexi, Bellilinea*	Yes, scafffold 10483	Yes, bin-25	0.3	0.1	No
OTU-32	*Spirochaetae, Exilispira*	Yes, scaffolds 5322 and 20220	No	0.2	1.1	No
OTU-33	*OP10/Armatimonadetes*_Gp7, OS Type L	Yes, scaffold 62246	No	0.2	0.0	No
OTU-34	*Chlorobi, Ignavibacteriaceae*	Yes, scaffolds 10292 and 68906	Maybe, bin-24	0.2	0.2	No
OTU-35	*Spirochaetae, Brevinema*	No	No	0.2	0.3	No
OTU-36	Acidobacterium, OPB3	Yes, scaffolds 1343 and 2292	Yes, bin-20	0.2	0.0	No
OTU-37	*Verrucomicrobia*, uncultured	Yes, scaffold 25955	No	0.2	0.0	No
OTU-38	*Chlorobi, “Ca*. Thermochlorobacter aerophilum”	Yes, scaffold 761	Yes, bin-21	0.2	2.2	No
OTU-39	*Chloroflexi, Anaerolineae*	Yes, scaffold 145123	No	0.2	0.1	No
OTU-40	*Deltaproteobacteria, Desulfarculales*, uncultured	Yes, scaffold 112567	No	0.2	0.0	No
OTU-41	*Spirochaetae, Spirochaeta*	Yes, scaffold 19076	No	0.2	0.1	No
OTU-42	*Acetothermia, uncultured*	(Yes, scaffold 169700)^*^	No	0.1	0.0	No
OTU-43	*Spirochaetae, Leptospiraceae, uncultured*	(Yes, scaffold 205406)^*^	No	0.1	0.0	No
OTU-44	*Deltaproteobacteria, Synthroporhabdus-like*	Yes, scaffolds 233149 and 241554	No	0.1	0.0	No
OTU-45	*Chlorobi, Ignavibacteriaceae*	Yes, scaffolds 104127 and 141772	Maybe, bin-24	0.1	0.0	No
OTU-46	*Elioraea* sp.	Yes, scaffold 23894	Yes, bin-22	0.1	0.0	No

* *based on phylogenetic analysis, no overlap of sequences*.

a *Klatt et al. (2011)*.

b *no metagenomic bin, but related sequences recruited by reference genomes*.

#### Aminicenantes (OP8)

The *Aminicenantes* (Candidate phylum OP8) was represented by only a single taxon, OTU-13, and its corresponding metagenomic Bin-13, which contains a 1,497-bp 16S rRNA gene sequence (Table 1, Table S1). Notably, OTU-13 amplicon sequences were found exclusively in the undermat community (Table S1). Although the iTag sequence shared 99% nt identity to the uncultured *Aminicenantes* bacterium clone OPB95 obtained from a Yellowstone hot spring (AF027060, Hugenholtz et al., 1998b), the full-length sequence showed only 95% nt identity to that sequence. No isolated bacterium shares more than 88% nt identity with this uncultured organism. 16S rRNA gene sequence surveys indicated that members of the *Aminicenantes* are ubiquitously present in many different habitats and across many environmental parameters (temperature, salinity, and oxygen tension) (Farag et al., 2014). They usually represent only a small fraction (<1%) of microbial communities, but have been found to be more abundant in anoxic environments (Farag et al., 2014).

#### Planctomycetes

Five abundant iTag OTUs were identified as belonging to members of the phylum *Planctomycetes* (Table S1), the very abundant OTU-14 (1,260 reads), as well as four less abundant OTUs (OTUs-16, 19, 49, and 51, Table S1). Twelve additional *Planctomycetes* sequences were found in very low abundance (Table S1).

Bin-14 contained a partial genome for *Planctomycetes* member OTU-14 and was identified based on the corresponding full-length 16S rRNA sequence as well as nineteen phylogenetic marker genes (Table 3, Figure S1). An uncultured hot spring-associated bacterium from a neutral 61°C geothermal hot-spring mat in Tibet, clone TP5, was identified as closest relative (EF205581, 99%, Lau et al., 2009). The microaerophilic, facultatively anaerobic, thermophilic *Planctomycetes* strain, *Thermogutta terrifontis* strain R1^T^ (KC867694, Slobodkina et al., 2014), with 90% sequence identity, is the most closely related isolated relative (Table 1). Based on the number of phylogenetic marker genes present in the metagenome bin, and because of the large sizes of available *Planctomycetes* genomes (3.8–9.7 Mb for those in JGI/IMG as of December 2015), we expect the 1.87-Mb bin to represent no more than 60% of the genome. The presence of the iTag sequences for this OTU almost exclusively in the undermat sample (a single read was found in iTag analysis of upper green layer; Table 5, Table S1) suggests that this organism lives exclusively in the orange-colored undermat and possibly in its deeper regions below 3 mm, where mainly anoxic conditions occur and persist (Nübel et al., 2002; Jensen et al., 2011).

Bin-23 was also identified as derived from a member of the *Planctomycetes*, but could not be directly affiliated with any iTag sequence(s) due to absence of an rRNA sequence in the bin (Table 3).

#### Acidobacteria

Thirteen OTUs representing four different members of the *Acidobacteria* were identified in the Mushroom Spring undermat community, and two of them were abundant with >100 reads (Table S1). OTU-17 was a member of group 4 of the *Acidobacteria* and was identified as *Cab. aerophilum* (Tank and Bryant, 2015a,b). Bin-16 (Table 3) contained a partial genome for this unique microaerophilic, chlorophototrophic member of the phylum *Acidobacteria*, which was first identified in the phototrophic mats of Mushroom and Octopus Spring and corresponds to the OS Type D sequences from earlier studies (Ward et al., 1990, 1992; Bryant et al., 2007; Tank and Bryant, 2015a,b).

OTU-36, as well as the less abundant OTU-72, were members of *Acidobacteria* group 3 and were identified as *Solibacter*-like organisms. Bin-20 was associated with OTU-36 by the presence of a 16S rRNA-containing scaffold as well as by the presence of six phylogenetic marker genes (Table 3). All six phylogenetic marker genes indicated an affiliation with the *Acidobacteria* and four of them specifically with the candidate species, “*Ca*. Solibacter usitatus” (Challacombe et al., 2011). Phylogenetic analysis supported the affiliation and placed the sequence in subgroup 3 of the *Acidobacteria*, closely related to Yellowstone clone OPB3 (98%, AF027004, Hugenholtz et al., 1998b) and “*Ca*. Solibacter usitatus” Ellin6076 as the closest named relative (Table 1, Figure S1). The low number of phylogenetic marker genes indicates that this member of the *Acidobacteria* has a large genome, only a part of which is included in the metagenomic bin. This correlates well with the fact that “*Ca*. Solibacter usitatus” Ellin6076 has an exceptionally large, 9.97-Mb genome (Challacombe et al., 2011).

The fourth member of the phylum *Acidobacteria* corresponded to a less abundant OTU (OTU-61, 70 reads = 0.1%) and was represented by two partial 16S rRNA sequences in the metagenome. These sequences and the represented uncultured organisms were affiliated with OS Type K sequences from previous studies (Table 4, Ward et al., 1992; Weller et al., 1992).

#### Proteobacteria

Four abundant OTUs were affiliated with the phylum *Proteobacteria* by the RDP classifier, one of which was misidentified as *Proteobacteria* and rather represents a *Brevinema*-like member of the *Spriochaeta* (OTU-35), two of which were *Deltaproteobacteria* (OTUs-40 and 44), and one of which was an *Alphaproteobacterium* (OTU-46). Twenty-nine additional, low-abundance OTUs were affiliated with *Proteobacteria* by RDP classifier (Table S1). Sequences for 16S rRNAs of two *Alpha*-, two *Beta*- and one *Delta-Proteobacteria* were found in the metagenome (Figure S1C). The abundant deltaproteobacterial sequence (OTU-44) was closely affiliated to a sequence obtained in a previous metagenome study (Figure S1C, Klatt et al., 2013a). Although the *Deltaproteobacteria* are commonly known to include members with sulfate-reducing metabolism, and sulfate-reduction has been shown in the microbial mat at Mushroom Spring (Dillon et al., 2007), deltaproteobacterial *dsrAB* genes were not identified in this nor any previous study. No metagenomic bin was affiliated with a *Deltaproteobacterium*.

The abundant *Alphaproteobacterium* (OTU-46) was identified as an *Elioraea* sp. within the *Rhodospirilliales*, which corresponds to OS Type O obtained in previous studies (Figure S1C, Table 4, Ward et al., 1992). The corresponding partial genome (Bin-22, Figure 4, Table 3) as well as the genome for the closest relative, *Elioraea tepidiphila* DSM 17972 (NCBI acc. no. NZ_KB899965.1), contain genes for anoxygenic photosynthesis. Although chlorophototrophy has not been described for *Elioraea tepidiphila* (Albuquerque et al., 2008), the ability to synthesize BChl *a* is predicted for the OTU-46 population in the undermat community. A BChl *a* containing strain, “*Candidatus* Elioraea thermophilum,” was isolated from the mat, which shares 99.8% and 99.2% sequence identity with the 16S rRNA sequences from the metagenome and amplicon study, respectively (Figure S1C, Tank et al., in press). A low abundance *Alphaproteobacterium* sequence (OTU-121, 16 reads) was identified as belonging to a *Roseomonas/Rhodovarius*-like organism, for which an isolate has been obtained from Mushroom Spring and which has tentatively been named “*Candidatus* Roseovibrio tepidum” (Figure S1C, Tank et al., in press). The isolate exhibits BChl *a* autofluorescence suggesting a phototrophic lifestyle, which is further strengthened by the presence of low coverage, unidentified alphaproteobacterial *pufLM* sequences in the metagenome (scaffold JGI24185J35167_1024732, genes 2 and 3, 20 × coverage). Only a single described *Roseomonas* sp., *R. aestuarii*, has been reported to produce BChl *a*, but no *pufLM* sequences are available for that isolate (Venkata Ramana et al., 2010). Furthermore, two low-abundance OTUs (OTUs-101 and 154) showed the same phylogenetic affiliation (*Hydrogenophilius* sp., *Betaproteobacteria*) as OS type G from previous studies (Ward et al., 1990, 1992). The OS Type R sequence (NCBI acc. no. U46750, unpublished) represented an unidentified *Betaproteobacterium* and a similar, low-abundance iTag sequence (OTU-172) was detected in this study (Table 4, Figure S1C).

#### Bacteroidetes-Chlorobi

The RDP classifier identified twenty and eight different OTU sequences belonging to members of the phyla *Chlorobi* and *Bacteroidetes*, respectively. Seven OTUs affiliated with the *Chlorobi* were abundant with read numbers >100, and one was very abundant with >1,000 reads (Table S1). However, the most abundant “*Chlorobi*” sequence (OTU-10) was mis-classified and represents an Thermotogae/EM3 group member (see above, Table 1, Figure 5B). The other abundant *Chlorobi* sequences were affiliated with the proposed family *Thermochlorobacteriaceae* (OTU-38) (Liu et al., 2012), “*Chlorobi* lineage 5” = “OPB56 group” (OTUs 24, 27, and 29) (Iino et al., 2010; Hiras et al., 2015) and “*Chlorobi* lineage 2” = “SM1H02 group” (OTUs 34 and 45) (Iino et al., 2010; http://www.arb-silva.de/browser/ssu-121/AY555793, named after clone SM1H02, Genbank acc. no. AF445702). Bin-19 (Table 3) was identified as a partial genome representing OTU-24, a representative of OPB56, a subgroup of the *Chlorobi* with predicted chemoheterotrophic lifestyle that was first detected in YNP (Hugenholtz et al., 1998b; Hiras et al., 2015, Table 3). A low abundance OTU in the OPB56, OTU-262, was identified as a probable representative of the OS Type F sequences from previous studies (Table 4, Ward et al., 1990, 1992). The first aerobic, phototrophic member of the *Chlorobi*, “*Ca*. Tcb. aerophilum,” which belongs to the proposed family *Thermochlorobacteriaceae* and was identified in the upper green layer of the microbial mat by previous metagenomic analyses (Liu et al., 2012), is represented by OTU-38 (Table 1), and was identified as OS Type E in previous studies (Ward et al., 1990, 1992; Ferris et al., 1996b). Bin-21 is derived from this novel phototroph (Table 3) and supports its characterization as a chlorophototroph that synthesizes type-1 reaction centers and chlorosomes, similar to cultivated relatives among the green sulfur bacteria, but which is otherwise very different physiologically. “*Ca*. Tcb. aerophilum” is proposed to be an aerobic photoheterotroph that cannot oxidize sulfur compounds, cannot fix N_2_, and does not fix CO_2_ (Liu et al., 2012).

Bin-24 (Table 3) does not contain a 16S rRNA sequence, but was affiliated with a putative member of the *Bacteroidetes-Chlorobi* group based on phylogenetic marker genes. It is most closely related to heterotrophic members of the *Chlorobi*, in the family *Ignavibacteriaceae* (Liu et al., 2012; Kadnikov et al., 2013) and is presumably affiliated with OTUs-34 or 45 in the *Chlorobi* Lineage 5/group SM1H02 (Figure 5B). All genes needed for dissimilatory sulfate reduction are present in the partial genome and indicate that this organism is putatively the first sulfate-reducing member of the *Bacteroidetes-Chlorobi* group. These results will be described in detail elsewhere (Thiel et al., in preparation). The OS Type M sequences obtained in previous studies (Ward et al., 1992) are affiliated with OTU-34 as well as with two partial 16S rRNA sequences from the metagenome (Table 4) within the SM1H02 (*Chlorobi* Lineage 2) group.

Only low abundance OTUs were affiliated with the *Bacteroidetes* (Table S1). Many of them were closely related to clone sequences obtained in a previous undermat study, and some also represented partial 16S rRNA sequences from the metagenome (Figure 5B, Klatt et al., 2013a). *Schleiferia thermophila*, a strain of which has been isolated from Octopus Spring microbial mats (Thiel et al., 2014a), was not detected in this study.

#### Deinococcus-Thermus/Thermi

Of two different members of the phylum *Thermi* identified in this study, only *Meiothermus* sp. was abundant in the undermat community (OTU-21, 656 reads), whereas sequences of *Thermus* spp. were only present in low numbers in the iTag study (Table S1, Figure S1C). Members of both genera have been isolated from these mat communities (Brock and Freeze, 1969; Ward et al., 1997; Thiel et al., 2015). OTU-21 was identified as a relative of *Meiothermus ruber*, a member of which, strain A, has previously been isolated from an enrichment culture originally obtained from the microbial mats at Octopus Spring and whose genome has been sequenced (Thiel et al., 2015). Tetranucleotide frequency-based binning of contigs >10 kb led to a 1.3-Mb partial genome (Bin-18, Table 3) for this moderately thermophilic, aerobic, and heterotrophic bacterium. The *Meiothermus* sp. 16S rRNA sequences obtained from the metagenome share 96.7% nt sequence identity with *M. ruber* strains A and DSM1279^T^. Sequences of Bin-18 shared 84.5% (±4.5%) with the *M. ruber* strain A genome and 84.2% (±4.5%) with *M. ruber* DSM1279^T^. Although the (partial) genome sequences of the isolate and the metagenome bin clusters overlap, some separation was visible when the sequences of both organisms were included in the binning analyses (data not shown).

#### Archaea

Although methanogenesis has been demonstrated in several mats of alkaline siliceous hot springs, including Mushroom Spring (Ward, 1978; Sandbeck and Ward, 1982), and methane has been shown to accumulate in the water above the Mushroom Spring mat in darkness (Kim et al., 2015), iTag sequencing only identified a few partial 16S rRNA sequences as potentially derived from methanogenic *Archaea* (OTUs-143, 151, 162, 192, and 244; ≤ 11 reads = ≤ 0.01%, Table S1). Phylogenetic analysis confirmed affiliation to the *Euryarchaeota* for four of them (OTUs-143, 151, 162, and 192, Figure S1A) and three of the sequences were detected in a cloning experiment from a previous study (Klatt et al., 2013a); thus, methanogenic archaea seem to be present in the mat over time, although in very low abundance. One OTU, OTU-151 with 10 reads but no representative sequence in the metagenome, shows high similarity (99% nt id) with the 16S rRNA sequence of the methanogenic archaeon *Methanothermobacter thermoautotrophicus*, strains of which have been isolated from these mats previously (former *Methanobacter thermoautotrophicum*; Sandbeck and Ward, 1982). Further, a single, low coverage *mcrA* gene encoding a methyl-coenzyme M reductase alpha subunit was present in the metagenome (JGI24185J35167_11200021, 7 × coverage) possibly indicating methanogenic metabolism in at least one of the archaeal mat members. Two slightly more abundant 16S rRNA sequences affiliated with ammonia-oxidizing *Archaea* were detected (Table S1). One (OTU-60, 72 reads) was related to “*Candidatus* Nitrosocaldus yellowstonii,” which was also identified in an enrichment culture from Octopus Spring mat in previous studies (De La Torre et al., 2008). The other, OTU-67 represents a member of a putatively novel archaeal phylum/division, related to “*Candidatus* Caldiarchaeum subterranum” (Nunoura et al., 2011). Another less abundant iTag sequence, similar to that of an archaeal 16S rRNA sequence recovered from the undermat metagenome previously (Klatt et al., 2013a), was also detected in the iTag analysis (OTU-125, 15 reads), but not in the metagenome of this study (Figure S1A). None of the metagenomic bins could be identified as belonging to *Archaea*, and only a few contigs with low coverage values, showed high identities to known archaeal sequences. Thus, our metagenomic and 16S rRNA gene amplicon studies indicate a very low abundance of *Archaea*, of which sequences related to ammonia-oxidizing *Archaea* seem to be more abundant than possible methanogenic *Archaea*. The low abundance of archaeal sequences is consistent with the low relative abundance of archaeal lipids in previous studies, which had been discussed to be related to the energy flows through the trophic structure of the community (Ward et al., 1989).

#### Firmicutes

Although *Anoxybacillus* spp. are common members of cyanobacterial enrichment cultures from these environments (e.g., Nowack, 2014; Olsen et al., 2015; Tank and Bryant, 2015b), no evidence for this organism was found in the metagenome nor the iTag analysis. Twenty-four OTUs were classified as belonging to members of the *Firmicutes*, of which two (OTUs-251 and 255) were predicted to be *Bacillus* sp.; however, they shared highest sequence similarity to the type strains of *Syntrophothermus lipocalidus* and *Acetomicrobium faecale* (both clostridia). None of the 16S rRNA genes retrieved from the metagenome could be affiliated with the *Firmicutes*. In addition, none of the metagenomic scaffolds were affiliated with *Anoxybacillus* spp. No sequence from an *Anoxybacillus* sp. was identified by BLASTn analysis of the metagenome using the partial genome sequence obtained from the *Anoxybacillus* sp. MT isolated from an enrichment culture from Octopus Spring (Thiel et al. in prep), nor the “phylogenetic distribution of genes by BLAST percent identities” tool implemented in the JGI/IMG website.

## Discussion

In this study we analyzed the orange undermat of the microbial mat community at 60°C in Mushroom Spring YNP by 16S rRNA gene amplicon and metagenomic sequencing. Only eight major organismal populations were identified in the upper green layer by genomic, metagenomic and metatranscriptomic analysis (Klatt et al., 2011; Liu et al., 2011). A higher diversity had been speculated to occur in the undermat community (Klatt et al., 2013a). In this study the undermat was found to be a highly diverse but uneven bacterial community, which could be related to the trophic structure associated with mat-decomposing organisms, as hypothesized to explain the variable abundances of lipid biomarkers (Ward et al., 1989) and 16S rRNA sequences (Ward et al., 1998). Out of 317 OTUs, the 15 most abundant ones represent 87% of all iTag sequences, and the single most abundant OTU comprises nearly half of all iTag reads. More than 44 abundant taxa, as defined by read numbers of >100 in the iTag analysis, were detected in the orange-colored undermat at Mushroom Spring. The phylum *Chloroflexi* displayed the highest diversity with nine abundant and 41 total taxon-specific 16S rRNA sequences (OTUs) found. All of the taxa found in the upper mat by Klatt et al. (2011) were also identified in the undermat.

In this study we analyzed the composition and diversity of the microbial community based on 16S rRNA gene sequences, which cannot easily be translated into species populations. However, relatively high 16S rRNA sequence diversity was found in this study, not only on the OTU level but particularly within the dereplicated iTags, which suggests that this microbial mat community is not simple. Previous observations that closely related cyanobacterial 16S rRNA sequences were differently distributed along environmental gradients (Ferris and Ward, 1997; Ramsing et al., 2000) prompted consideration of the Stable Ecotype Model of species and speciation (Cohan and Perry, 2007), which postulates that some microorganisms exist as ecological species occupying distinct niches (Ward, 1998; Ward and Cohan, 2005). Studies with more rapidly evolving protein-encoding loci led to the prediction of numerous ecotypes with identical or nearly identical 16S rRNA sequences (Ferris et al., 2003; Becraft et al., 2011, 2015; Melendrez et al., 2011). The existence of temperature- and light-adapted *Synechococcus* ecotypes has been demonstrated by obtaining representative strains and studying their temperature and light preferences as well as their genomes, (Allewalt et al., 2006; Nowack et al., 2015; Olsen et al., 2015). A similar microdiversity and existence of putative ecotypes is suggested by this study for members of the undermat community, and in particular for *Roseiflexus* spp., the most dominant member in the undermat. The presence of unique 16S rRNA genotypes in the undermat (this study) and at different temperatures (Ferris and Ward, 1997), supports this inference. In addition to the high diversity of OTUs within the phylum *Chloroflexi*, a high microdiversity was found for *Roseiflexus* spp. by the presence of 24 abundant and a total of 6,193 dereplicated *Roseiflexus* sp. iTag sequences, which is further supported by a preliminary analysis of *pufLM* amplicon sequence data (J. Wood and D. Ward, unpublished data).

The microbial mat as a living and active biological system has been shown to be constantly growing (Doemel and Brock, 1977). In this study we observed phototrophic taxa known from the upper layer in the undermat. Analyses of *psaA* sequences sampled in this metagenomic study suggest that the *Synechococcus* populations observed match species found in the upper mat and thus likely occur in the undermat as a consequence of burial. In contrast, similar analyses of *pufLM* sequences as well as oligotyping suggest that *Roseiflexus* populations in the undermat are a mixture of those found in the upper green mat layers and those uniquely found in the undermat (Table 5, Figure S2, Wood et al., unpublished). The detection of identical dereplicated iTag and oligotype sequences in both layers might indicate burial. However, the detection of oligotypes and dereplicated iTag sequences with higher relative abundance in the undermat strongly suggests the existence of putative ecotypes specifically adapted to niches in the undermat. Further it is important to note that specifically adapted ecotypes can be so closely related that they have the identical 16S rRNA gene sequence, and can only be detected using more rapidly evolving genes (Becraft et al., 2011, 2015). For other organisms, a greater relative abundance, or exclusive presence in the lower part of the mat, is indicated by the relative number of 16S rRNA gene amplicon reads between the upper layer and undermat samples. For example, *Pseudothermotoga* spp. OTU-2, *Armatimonadetes* member OTU-3, *Thermocrinis* spp. OTU-4, *Chloroflexi* members OTU-6, 9, and 15, as well as the *Atribacteria* member OTU-7, the *Aminicenantes* member OTU-13, and *Planctomycetes* member OTU-14, are found in much higher relative abundance in the undermat (Table 5, Table S1). Future transcriptomic studies will assess which of the detected populations correspond to the highest transcriptional activities based on gene expression. The presence of aerobic, microaerobic and anaerobic organisms detected in this study indicate a possible layered distribution along the steep and fluctuating oxygen gradient and shows that some oxygen is available during the day below a depth of 2 mm in the microbial mat, as previously suggested by microelectrode measurements (Revsbech and Ward, 1984; Nübel et al., 2002; Jensen et al., 2011). Whereas aerobic bacteria and facultative anaerobes are expected to live in the transition zone adjacent to the upper green layer, abundant anaerobic members of the undermat community, e.g., *Pseudothermotoga* sp. OTU-2 and *Atribacteria* member OTU-7 can be expected to be active members mainly in the community below a depth of 3 mm, where anoxic conditions are expected to persist throughout the day (Nübel et al., 2002; Becraft et al., 2011; Jensen et al., 2011). Despite the anaerobic lifestyle of sulfate reduction, *Thermodesulfovibrio* sp. OTU-8 was detected in higher abundance in the upper layer, which might indicate some degree of oxygen tolerance and diel activity patterns, i.e., primary sulfate-reducing activity under anoxic conditions in the afternoon or at night as measured by Dillon et al. (2007). An *Aminicenantes* (OP8) member (OTU-13), a *Planctomycetes* member (OTU-14) and an *Oscillochloris*-like chlorophototrophic member of the *Chloroflexi, “Ca*. Chloranaerofilum corporosum” (OTU-15) (Tank et al., in press) were exclusively detected in the undermat by iTag analysis, which suggests that they have an anaerobic lifestyle in the deeper layers of the undermat. However, “*Ca*. Chloranaerofilum corporosum” is expected to be a phototroph, and only a limited amount of light reaches deep into the undermat. Thus, a layered structure of the microbial community, as has been demonstrated in the upper green layer (Ramsing et al., 2000; Becraft et al., 2011), can only be hypothesized for the undermat at this time. Further studies are needed to determine the distribution of the members of the undermat community.

All seven chlorophototrophs identified in previous genomic and metagenomic studies of the upper green layer were also present in the undermat metagenome (Table 5; Klatt et al., 2011; Liu et al., 2011). *Roseiflexus* spp. and “*Candidatus* Roseilinea gracile” showed higher relative abundance in the undermat, whereas the other phototrophs are present in lower relative abundance in comparison to the upper green layer of the mat (Table 5, Table S1). Three additional phototrophic bacteria were detected in the microbial mat for the first time in this study (“*Candiatus* Chloranaerofilum corporosum” OTU-15, as well as two phototrophic *Alphaproteobacteria*, “*Candidatus* Elioraea thermophila” OTU-46, and “*Candidatus* Roseovibrio tepidum” OTU-121; Tank et al., in press). A total of sixteen phototrophic bacterial taxa representing six different phyla have now been detected in the Mushroom Spring microbial mat (Tank et al., in press). Additionally, the discovery of multiple organisms with genes encoding xanthorhodopsin raises new questions about the role of retinal-based phototrophy (retinalophototrophy; Bryant and Frigaard, 2006) or signaling in the undermat. This will be addressed in more detail elsewhere (Thiel et al., in preparation). The unidentified Cluster 8 previously detected in the upper layer metagenome was identified again here as OTU-10, an organism affiliated with the group EM3, which has tentatively been placed in the phylum *Thermotogae* (Reysenbach et al., 1994; Klatt et al., 2013a). The second unidentified heterotroph previously detected in the upper layer metagenome, Cluster 7 (Klatt et al., 2011), was identified as an *Armatimonadetes* member OTU-3. Due to a high microdiversity of this organism in the microbial mat sample, identification was only possible by a serendipitous finding of a closely related organism in an enrichment culture.

## Conclusions

In this study we analyzed the community composition and diversity of the orange-colored undermat of Mushroom Spring, an alkaline hot spring in YNP (WY, USA) by 16S rRNA gene amplicon and metagenomic analyses. Despite a long history of research on the microbial mats at Mushroom and Octopus Springs (Brock, 1967; Ward et al., 1998, 2012; Kim et al., 2015), these mats still harbor the potential for many novel discoveries. Members of the genus *Roseiflexus* dominated a fairly diverse but uneven microbial community, and metagenomic analysis identified several novel organisms with unusual traits. Many unidentified 16S rRNA sequences recovered from these environments in previous studies were detected and phylogenetically identified. Other organisms, which have been cultured from either Mushroom or Octopus Spring, were not detected, once again illustrating the inherent bias of untargeted cultivation experiments. A more detailed analysis of the metagenome, focusing on the metabolic potential of the mat members and their putative interactions, will be published elsewhere (Thiel et al., in preparation). Studies of microbial ecology, diversity, species evolution and interspecies interactions are still subjects of ongoing research with many open questions to be addressed. Comparisons of species in both upper and lower mat and a diel-transcriptomic analysis that will hopefully reveal gene expression activity within the undermat community that will allow us to distinguish between active and inactive members of the community defined in this study, and should provide information on the temporal pattern of gene expression in the undermat. Depth-dependent distributions of OTU populations that may represent putative ecotypes will also be addressed in future studies.

## Accession numbers

16S rRNA gene sequences of iTag OTUs as well as assembled clone sequences have been deposited in GenBank (Acc. nos. KU860141–KU860455 [iTag OTUs]; KX213895–KX214032 [clone OTUs]). Complete metagenome data are available in the Integrated Microbial Genomes with Microbiome Samples (IMG/M, https://img.jgi.doe.gov/) database, taxon object IDs 3300002493, 3300005452 and 2015219002.

## Author contributions

VT conducted sequence analysis after assembly for both amplicon and metagenome sequences, including phylogenetic analysis and phylogenetic marker genes analysis of metagenome bins. JW conducted initial tetranucleotide binning analyses, reference targeted mapping studies and contributed to discussion and manuscript. Sampling and DNA extraction from the hot-spring microbial mat and enrichment cultures was conducted by MO, who also wrote corresponding sections in the manuscript and contributed to the discussion of results. MT isolated and identified all cultures mentioned in the manuscript, contributed to writing the manuscript and discussing the results. CK conducted 16S rRNA cloning and sequencing from undermat samples from a previous time point, analyzed those sequences and contributed to manuscript and discussion. Sequencing, quality check, assembly and dereplication of amplicon and the metagenome was conducted by JGI staff. DW and DB planned the experiments, acquired funding, organized and led field excursions and provided scientific infrastructure. VT, DW, and DB wrote the manuscript.

## Funding

This study was partly funded by the Division of Chemical Sciences, Geosciences, and Biosciences, Office of Basic Energy Sciences of the Department of Energy through Grant DE-FG02-94ER20137. DB and DW additionally acknowledge support from the NASA Exobiology program (NX09AM87G). This work was also partly supported by the U. S. Department of Energy (DOE), Office of Biological and Environmental Research (BER), as part of BER's Genomic Science Program 395 (GSP). This contribution originates from the GSP Foundational Scientific Focus Area (FSFA) at the Pacific Northwest National Laboratory (PNNL) under a subcontract to DB. The nucleotide sequencing was performed as part of a Community Sequencing Program (Project CSP-411) and was performed by the U.S. Department of Energy Joint Genome Institute, which is supported by the Office of Science of the U.S. Department of Energy under Contract No. DE-AC02-05CH11231.

### Conflict of interest statement

The authors declare that the research was conducted in the absence of any commercial or financial relationships that could be construed as a potential conflict of interest.

## Supplementary material

Figure S1 **Phylogenetic tree based on 16S rRNA gene sequences showing the phylogenetic relationship between sequences obtained from the Mushroom Spring microbial undermat community and cultivated and uncultivated relatives in the phyla Armatimonadetes OP10, Aminicenantes OP8, Nitrospirae, Thermodesulfobacteria, Microgenomates OP11, Aquificae, SM2F11 and Archaea (A), Planctomycetes, Verrucomicrobia, Spirochaeta, Acidobacteria, Atribacteria OP9/JS1, Elusimicrobia and Cyanobacteria (B), and Proteobacteria, Thermotogae/EM3 and Thermi (C)**. The trees were generated based on the Maximum Likelihood method using the phyML software included in the ARB package. Percentage numbers on nodes refer to 100 bootstrap pseudoreplicates conducted. Only values >50% are shown. Bold sequences were obtained from Mushroom or Octopus Spring in this or previous studies. Red bold labels indicate sequences obtained in this study. Blue bold labels indicate “OS type” sequences from previous studies. OTU numbers shown refer to the most abundant OTU represented by the sequence. Only sequences with length >1,000 bp were used for phylogenetic calculations. Sequence length <1,000 bp are given in (gray) in the labels and corresponding sequences were added using the Parsimony method without changing tree topology.

Figure S2 **Entropy distribution in *Roseiflexus*-like 16S rRNA gene amplicon sequences in the combined dataset (A), and the undermat (B), and upper layer (C)**. The difference in the entropy figures from upper layer **(C)** and undermat **(B)** analyzed separately, specifically the considerably lower entropy at pos. 104 and 109 in upper layer, are indicative of a lower abundance of two oligotypes in the upper layer, namely “CCCCGCGTGC” (2.13% in undermat, 0.19% in upper layer) and “CCCCGCGGGC” (1.02 vs. 0.21%) (Table S2).

Table S1 **OTUs obtained from 16S rRNA V4 iTag sequencing**. Read numbers, relative abundance and number of total and abundant dereplicated iTag sequences are stated. Classification are based on RDP classifier.

Table S2 **Read counts and relative abundance of the 23 most abundant *Roseiflexus*-like oligotypes in undermat and upper layer as determined in the combined amplicon dataset (>100 reads total)**.
