# Supplementary figures and images for: The Dark Side of the Mushroom Spring Microbial Mat: Life in the Shadow of Chlorophototrophs. I. Microbial Diversity Based on 16S rRNA Gene Amplicons and Metagenomic Sequencing
Source: Front Microbiol. 2016 Jun 17;7:919. doi: 10.3389/fmicb.2016.00919 (PMC4911352; doi:10.3389/fmicb.2016.00919)

Figure S1(A)

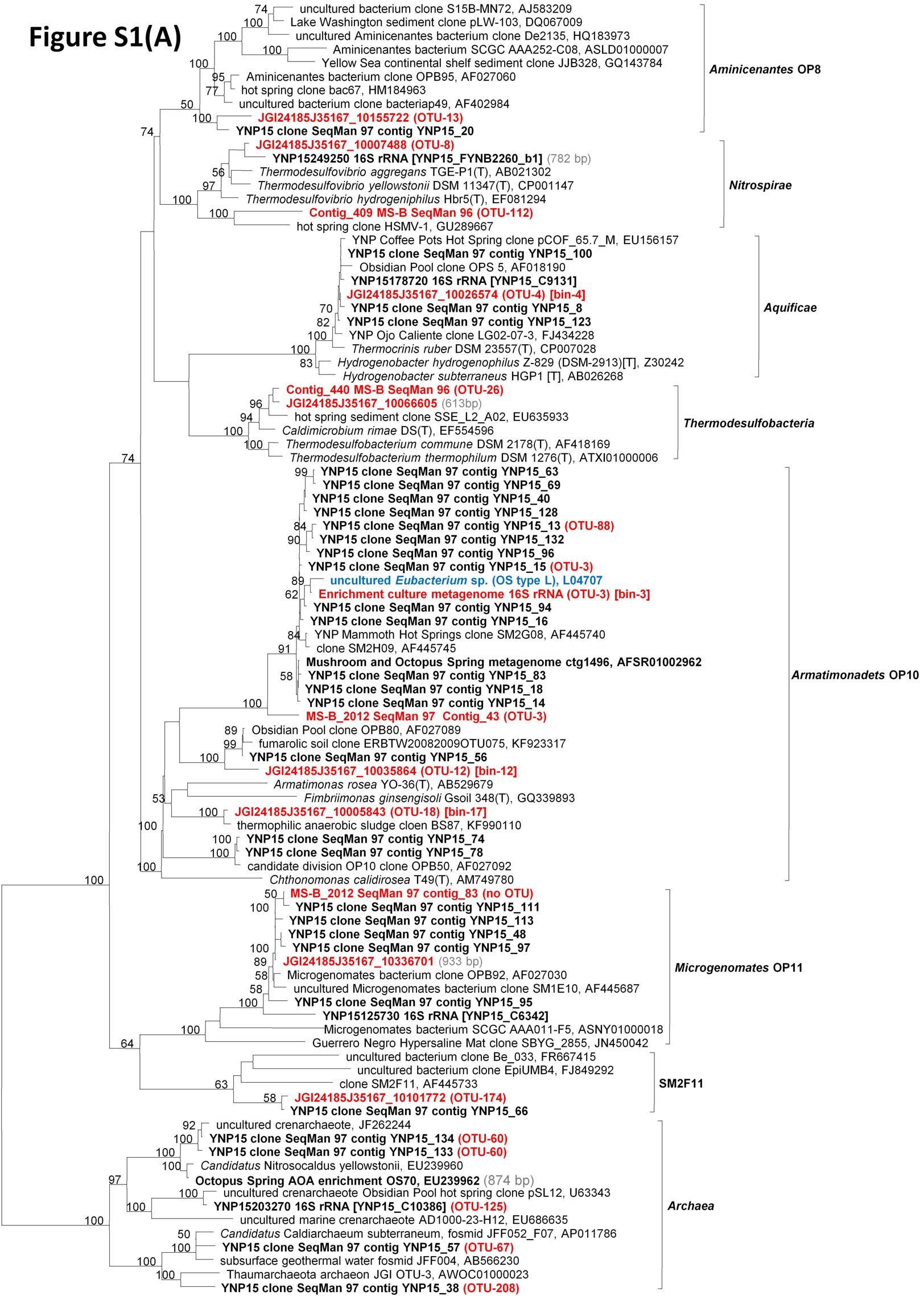

Figure S1(B)

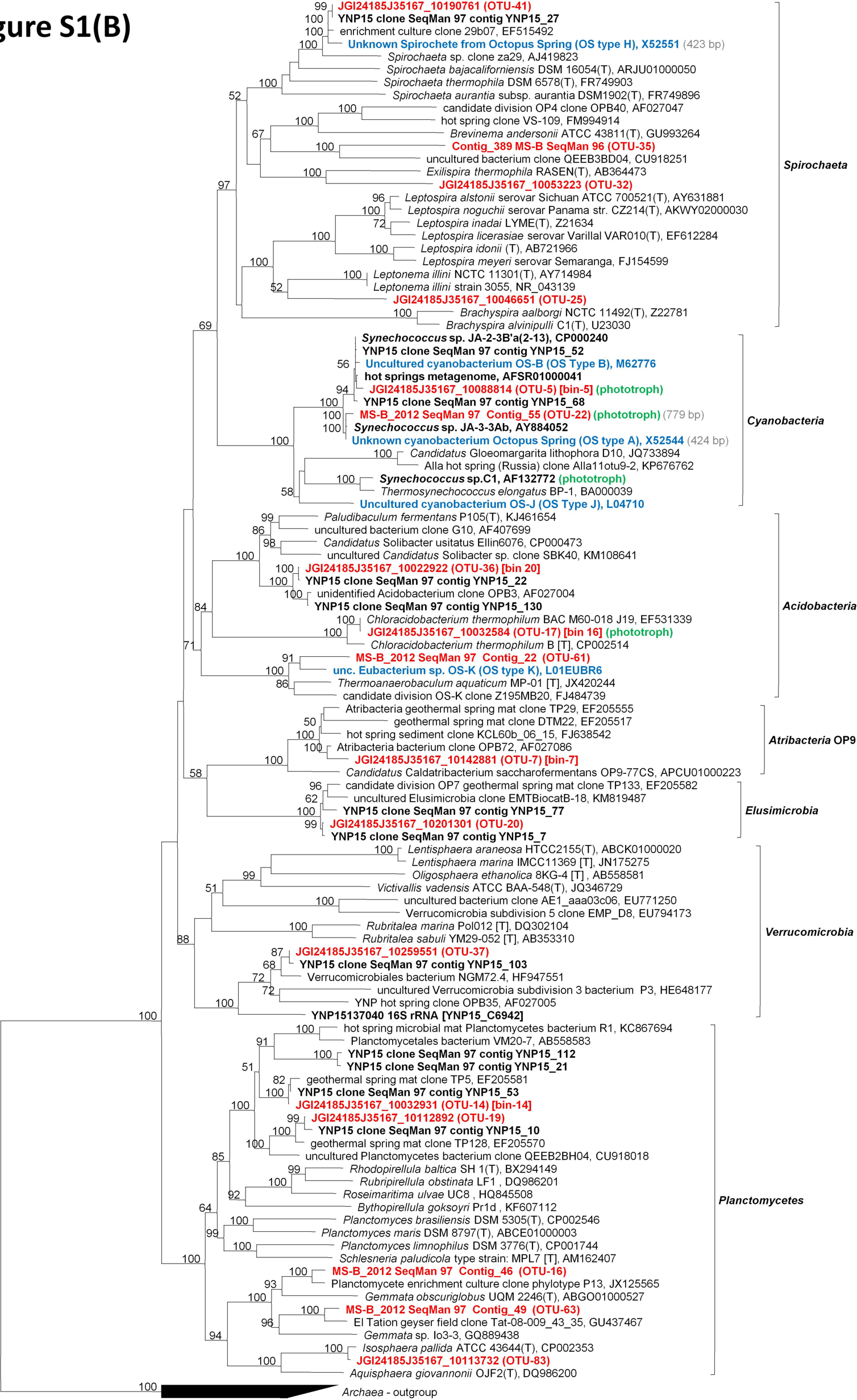

Figure S1(C)

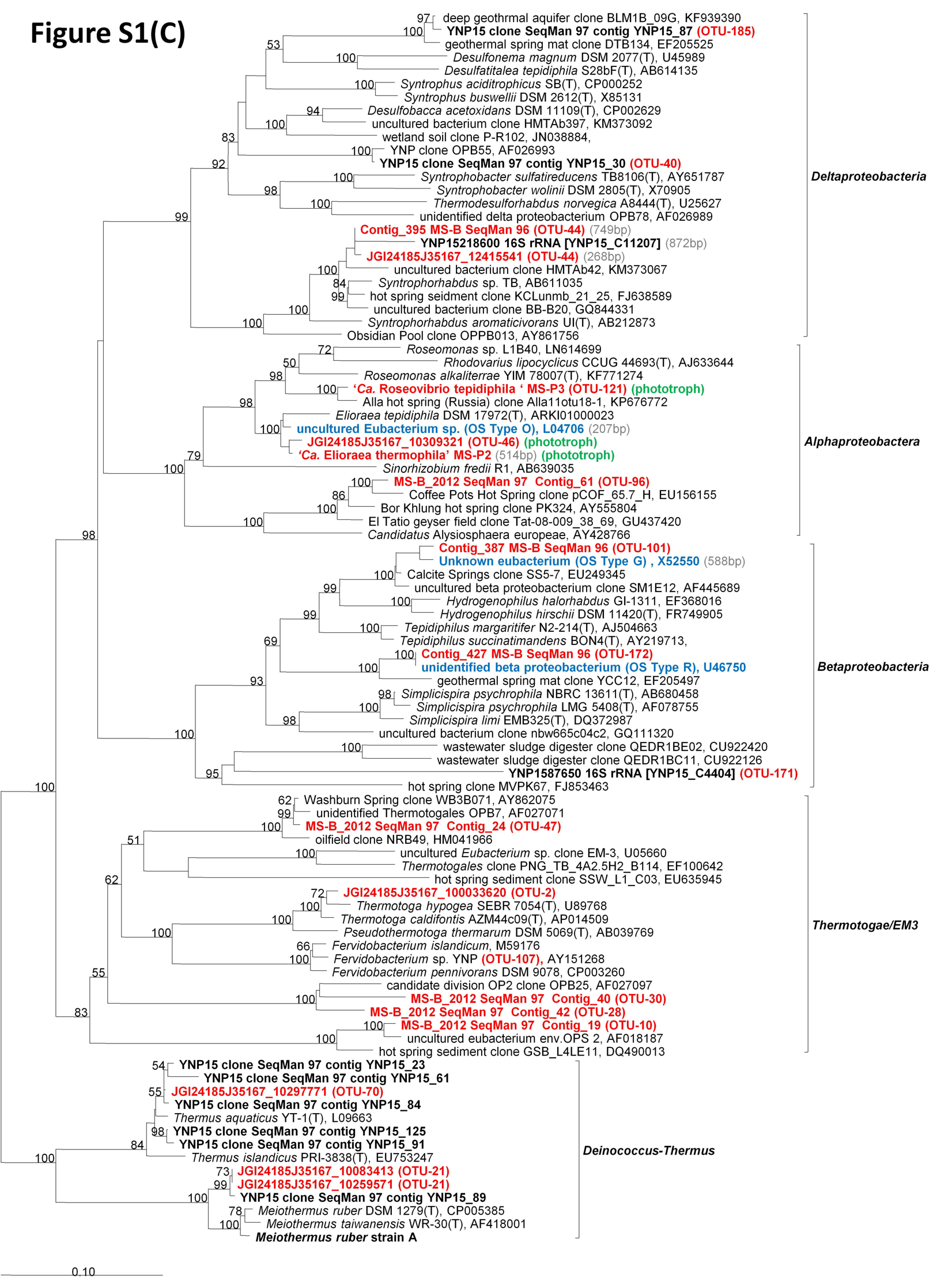

Supplement: Figure S1 — Phylogenetic tree based on 16S rRNA gene sequences showing the phylogenetic relationship between sequences obtained from the Mushroom Spring microbial undermat community and cultivated and uncultivated relatives in the phyla Armatimonadetes OP10, Aminicenantes OP8, Nitrospirae, Thermodesulfobacteria, Microgenomates OP11, Aquificae, SM2F11 and Archaea (A), Planctomycetes, Verrucomicrobia, Spirochaeta, Acidobacteria, Atribacteria OP9/JS1, Elusimicrobia and Cyanobacteria (B), and Proteobacteria, Thermotogae/EM3 and Thermi (C). The trees were generated based on the Maximum Likelihood method using the phyML software included in the ARB package. Percentage numbers on nodes refer to 100 bootstrap pseudoreplicates conducted. Only values >50% are shown. Bold sequences were obtained from Mushroom or Octopus Spring in this or previous studies. Red bold labels indicate sequences obtained in this study. Blue bold labels indicate “OS type” sequences from previous studies. OTU numbers shown refer to the most abundant OTU represented by the sequence. Only sequences with length >1,000 bp were used for phylogenetic calculations. Sequence length <1,000 bp are given in (gray) in the labels and corresponding sequences were added using the Parsimony method without changing tree topology. [file Image1.pdf]

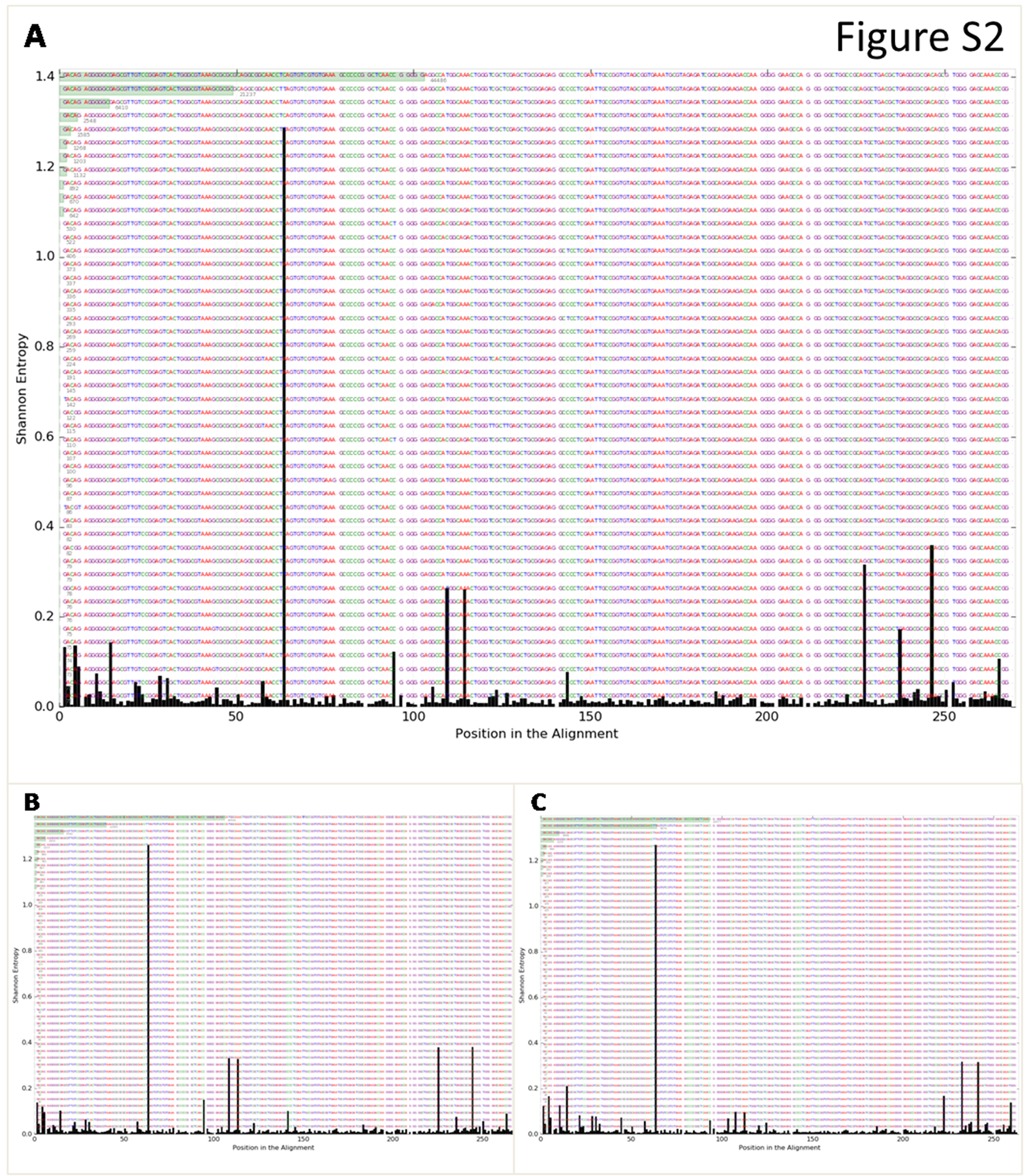

Supplement: Figure S2 — Entropy distribution in Roseiflexus-like 16S rRNA gene amplicon sequences in the combined dataset (A), and the undermat (B), and upper layer (C). The difference in the entropy figures from upper layer (C) and undermat (B) analyzed separately, specifically the considerably lower entropy at pos. 104 and 109 in upper layer, are indicative of a lower abundance of two oligotypes in the upper layer, namely “CCCCGCGTGC” (2.13% in undermat, 0.19% in upper layer) and “CCCCGCGGGC” (1.02 vs. 0.21%) (Table S2). [file Image2.tif]
