# Supplementary material for: The Dark Side of the Mushroom Spring Microbial Mat: Life in the Shadow of Chlorophototrophs. I. Microbial Diversity Based on 16S rRNA Gene Amplicons and Metagenomic Sequencing
Source: Front Microbiol. 2016 Jun 17;7:919. doi: 10.3389/fmicb.2016.00919 (PMC4911352; doi:10.3389/fmicb.2016.00919)
Supplement: Table S2 — Read counts and relative abundance of the 23 most abundant Roseiflexus-like oligotypes in undermat and upper layer as determined in the combined amplicon dataset (>100 reads total). [file DataSheet2.docx]

**Table S2 | Read counts and relative abundance of the 23 most abundant**

**Roseiflexus-like oligotypes in undermat and upper layer as determined in**

**the combined amplicon dataset (>100 reads total).**

|  | **MS-under** | | **MS-upper** | |
| --- | --- | --- | --- | --- |
| **Oligotype** | read counts | rel. abundance [%] | read counts | rel. abundance [%] |
| CCCTACGGGC | 40,492 | 53.524 | 18,624 | 47.823 |
| CTCTACGGGC | 15,358 | 20.301 | 12,605 | 32.367 |
| CACTACGGGC | 6,622 | 8.753 | 2,185 | 5.611 |
| CCCTACGGGA | 2,809 | 3.713 | 1,032 | 2.650 |
| CTCTACGGAC | 462 | 0.611 | 1,670 | 4.288 |
| CTCTACGGGA | 1,043 | 1.379 | 682 | 1.751 |
| CCCCGCGTGC | 1,614 | 2.133 | 72 | 0.185 |
| CCCTACGTGC | 1,277 | 1.688 | 321 | 0.824 |
| CACTACGTGC | 960 | 1.269 | 131 | 0.336 |
| CCCCGCGGGC | 774 | 1.023 | 81 | 0.208 |
| CCTCGCGTGC | 545 | 0.720 | 125 | 0.321 |
| CCTCGCGGGC | 610 | 0.806 | 51 | 0.131 |
| CACTACGGGA | 490 | 0.648 | 108 | 0.277 |
| CCCTACGGAC | 147 | 0.194 | 394 | 1.012 |
| CTCTACGTGC | 324 | 0.428 | 148 | 0.380 |
| TACTACAGGC | 267 | 0.353 | 28 | 0.072 |
| CACCGCGTGC | 214 | 0.283 | 28 | 0.072 |
| CCCTACGGGG | 142 | 0.188 | 27 | 0.069 |
| TCCTATGGGC | 31 | 0.041 | 118 | 0.303 |
| CATCGCGGGC | 138 | 0.182 | 4 | 0.010 |
| CTCTACGGAA | 36 | 0.048 | 81 | 0.208 |
| TACTACGGGC | 83 | 0.110 | 24 | 0.062 |
| CCCTACGTGA | 84 | 0.111 | 22 | 0.056 |

**Supplements. Material and methods.**

***Sample collection.*** The samples derive from a chlorophototrophic microbial mat in one of the effluent channels of the siliceous and slightly alkaline Mushroom Spring in Yellowstone National Park, WY (USA) [GPS position 44.539/-110.798]. The samples were collected under Yellowstone National Park collection permit YELL-00129, on August 10th, 2011 at 12:00 PM at 60°C degree water temperature using a #4 cork borer. The microbial mat is made up of a 1-2 mm green upper layer, which consists of different chlorophototrophic bacteria, and an orange-colored undermat layer (see **Figure 1**). The upper 2 mm of the sample containing the green layer was removed and stored separately, and the orange undermat sample (3-5 mm) was immediately frozen in liquid nitrogen. The samples were kept at -80°C until required.

***DNA extraction.*** Genomic DNA was isolated from five independent bacterial undermat samples using an enzymatic cell lysis protocol followed by phenol extraction. The frozen mat was thawed, resuspended in 100µL medium DH (Castenholz's medium D, Castenholz, 1969), and homogenized with a sterile mini-pestle in 2-ml screw cap collection tube. Cell lysis was initiated by incubating the sample for 45 min incubation at 37°C with 200 µg lysozyme ml^–1^. After a first microscopic check, SDS (1% w/v end-concentration) and Proteinase K (200 µgml^–1^) was added, and the samples were incubated at 50°C (50 min). Genomic DNA was extracted using phenol (pH 8.0) buffered with TE (10mM Tris-HCl, 1mM EDTA, pH 8), phenol-chloroform-isoamylalcohol (25:24:1) and a chloroform:isoamylalcohol (24:1) purification steps. DNA was precipitated overnight by addition of 0.1 volume 3M Na-acetate (pH 5.2), and 2.5 volumes 100% ethanol at -20°C. After pelleting in a microcentrifuge (18,000×*g*, 4°C, 30min), washing with 70% (v/v) ethanol and resuspension in TE buffer, the DNA was treated with RNase following the 'RNase I DNA Prep Clean up protocol' from JGI Sample Guidelines [http://jgi.doe.gov/collaborate-with-jgi/pmo-overview/protocols-sample-preparation-information/]. Due to low DNA concentration in each replicate, DNA extracts from the five parallel mat samples were pooled before sequencing to allow whole metagenome and amplicon sequencing from the same sample. Previous sampling of replicates have shown that the microbial community in cores from a localized region were very similar (Liu et al. 2011) and our primary goal was to determine the major players in the undermat community rather than study the distribution of microdiversity as a function of gradients.

***iTag sequencing at JGI.*** Bar-coded sequencing of variable regionV4 amplicons (primers 515F and 806R) of the 16S rRNA gene was conducted at JGI (JGI Project ID: 1031281, GOLD ID: Gp0061124) on an Illumina MiSeq instrument. Sample preparation was performed on a PerkinElmer Sciclone NGS G3 Liquid Handling Workstation utilizing the HotMasterMix amplification kit and custom amplification primers targeting the V4 region of the 16S rRNA gene of 5 PRIME. Primers also contained the Illumina sequencing adapter sequence and a unique barcode index sequence specific to each well on the plate, which allows for multiplexing of prepared amplicons and direct sequencing of them. Prepared amplicon libraries were normalized and multiplexed into a single pool of amplicons for the entire plate. The prepared pool of 16S V4 rRNA gene amplicon libraries were quantified using the next-generation sequencing library qPCR kit of KAPA Biosystem and analyzed on a Roche Light Cycler 480 real-time PCR instrument. The quantified pool was loaded onto an Illumina MiSeq sequencer using the v3 reagent kit and a 2×300 indexed run protocol.

***iTag sequence analysis at JGI.*** The generated reads were analyzed using the iTagger amplicon analysis pipeline v1.1 [https://bitbucket.org/berkeleylab/jgi_itagger, written by Julien Tremblay (julien.tremblay@mail.mcgill.ca) and Edward Kirton (ESKirton@LBL.gov); Copyright (c) 2013 by the US DOE Joint Genome Institute but freely available for use without warranty under the same license as Perl itself. v1.1 was released 12/12/2013]. After quality control of the reads by contaminant filtering and primer trimming, pairs were merged. Sequences were again filtered and removed when the number of expected errors exceeded the threshold (at Error Rate = 0.25). Sequences were then dereplicated (i.e., clustered by 100% similarity) and their observed abundances counted. Dereplicated sequences were clustered into OTUs using USEARCH with a threshold of 97% nt sequence identity. Taxonomic classification was conducted using RDP Classifier and likely chimeric sequences were discarded using UCHIME.

***Diversity analysis.*** Richness was estimated based on the richness estimator Chao1 (S_chao1_=S_obs_+(no. of singletons^2^)/(2* no. of doubletons) (Chao, 1984). Diversity was assessed using the Simpson's Reciprocal Index ($D=\frac{\sum n(n-1)}{N(N-1)}$), which is more sensitive to evenness than the Shannon Diversity index. Evenness as a measure of the relative abundance of the different species making up the richness of a community was assessed and visualized by a log abundance plot and a rank abundance curve for 97% identity OTUs.

Microdiversity was assessed using the number of high abundant dereplicated sequences, and the 'oligotyping pipeline' (http://merenlab.org/projects/oligotyping/). *Roseiflexus*-like sequences for oligotyping were extracted from the quality checked and merged but not dereplicated 16S rRNA gene amplicon sequence datasets by aligning to the *Roseiflexus* sp. RS-1 16S rRNA gene (CP000686, downloaded as aligned sequence from greengenes.lbl.gov) using the QIIME align_seqs.py script (Caporaso et al. 2010a) and the PyNAST (Caporaso et al. 2010b) method. Aligned sequences were trimmed of uninformative columns and entropy analysis performed using scripts provided in the 'oligotyping pipeline' (http://merenlab.org/projects/oligotyping/). Number of components and nucleotide positions for oligotyping analysis were chosen based on the entropy analysis and purity scores of complete datasets and proposed oligotypes. Undermat and upper layer datasets were analyzed combined and separately.

***Library construction and metagenome sequencing at JGI.*** DNA (100ng) was sheared to 270bp by sonication (Covaris). The fragments were treated by end-repair, A-tailing, and ligation of Illumina compatible adapters (IDT, Inc.) using the KAPA-Illumina library creation kit (KAPA Biosystems). The prepared sample library was quantified using a next-generation sequencing library qPCR kit (KAPA Biosystem)and analyzed with a Roche LightCycler 480 real-time PCR instrument. The quantified sample library was prepared for sequencing on the IlluminaHiSeq sequencing platform utilizing a TruSeq paired-end cluster kit, v3, and the cBot instrument of Illumina to generate a clustered flowcell for sequencing. Sequencing of the flowcell was performed on the Illumina HiSeq 2000 sequencer using Illumina TruSeq SBS sequencing kits, v3, following a 2×150 indexed high-output run protocol.

***Metagenome assembly methods at JGI.*** Raw HiSeq Illumina metagenomic reads were screened against Illumina artifacts with a sliding window with a kmer size of 28and a step size of 1. Screened reads were trimmed from both ends using a minimum quality cutoff of 3, and reads with 3 or more Ns or with average quality score of less than Q20 were removed. In addition, reads with sequence length of <50 bp were removed. Trimmed, screened, paired-end Illumina reads were assembled using SOAPdenovo v1.05 (http://soap.genomics.org.cn/soapdenovo.html) at a range of Kmers (assembled kmers were:81,85,89,93,97,101). Default settings for all SOAPdenovo assemblies were used (options "-K 81 -p 32 -R -d 1"). Contigs generated by each assembly (6 total contig sets), were de-replicated using in-house Perl scripts. Contigs were then sorted into two pools based on length. Contigs smaller than 1800 bp were assembled using Newbler (Life Technologies, Carlsbad, CA) to generate larger contigs (flags: -tr, -rip, -mi 98, -ml 80). All assembled contigs larger than 1800 bp, as well as the contigs generated from the final Newbler run were combined using minimus 2 (flags: -D MINID=98 -D OVERLAP=80) (AMOS:, http://sourceforge.net/projects/amos). Read depths were estimated based on read mapping with the JGI in-house mapping program.

***Metagenome analysis using img/mer.*** The metagenome was automatically annotated and subsequently analyzed using the integrated metagenome comparative analysis system toolset, IMG/MER (Markowitz et al., 2014), implemented in the JGI Integrated Microbial Genomes (IMG) system. In addition to the annotation information, GC contents, coverage, and phylogenetic distribution of genes based on BLAST percentage identity (PhyloDist) were used. Partial 16S rRNA genes were identified by the pipeline implemented in JGI/IMG analyses and used for assessment of community composition and identification of metagnomic bins via BLASTn and phylogenetic analysis. They can be accessed and downloaded from the publicly available metagenome under IMG Submission ID 35167 (Taxon Object ID 3300002493).

***Binning.*** Metagenomic scaffolds/contigs were binned based on tetranucleotide frequency patterns using emergent self-organizing maps (ESOM). The tetranucleotide frequency of all contigs ≥5,000bp was calculated using AndersAndersson's perl script 'tetramer_freqs_esom.pl' (https://github.com/tetramerFreqs/Binning/blob/master/tetramer_freqs_esom.pl; Dick et al., 2009). Contigs were split into 5,000-bp segments, clustered into genomic bins by tetranucleotide frequency and visualized with Databionic-ESOM (http://databionic-esom.sourceforge.net) using parameters from Dick et al. (2009).

***Annotation of bins.*** In addition to the IMG/MER annotation, partial genomes (metagenome bins) were automatically annotated using the RAST (Rapid Annotation using SEED Technology) annotation server (Aziz et al., 2008; Overbeek et al., 2014).

***Phylogenetic affiliation using phylogenetic marker genes.*** Phylogenetic affiliation based on phylogenetic marker genes was assessed using AmphoraNet (http://pitgroup.org/amphoranet/; Kerepesi et al., 2014), the webserver implementation of the AMPHORA2 metagenomic workflow suite, which uses 31 universal bacterial phylogenetic marker genes for taxonomic affiliation of a genome sequence or estimation of the taxonomic composition of metagenome sequences.

***Phylogenetic analyses.*** Sequences of 16S rRNA genes were downloaded from metagenome datasets from Mushroom Spring undermat samples available at the JGI website (JGI IMG taxon IDs 2015219002, 3300002493, 3300005452). 16S rRNA sequences >1,000bp were used in calculation of phylogenetic trees directly. Partial sequences were assembled using DNAStar Lasergene 12 SeqMan Pro software. Sequences derived from IMG taxon ID 3300002493, which were derived from the same DNA as the iTag sequences in this study, were primarily used. They were assembled using 97% sequence identity and used in the calculation of phylogenetic trees if they exceeded 1,000 bp (labeled in the tree "**MS-B 2012 SeqMan 97% contig_xx**"). If no long sequences were obtained from the ID 3300002493 metagenome, sequences obtained from IDs 3300002493 and 3300005452 (derived from a 2014 undermat sample) were jointly assembled using 96% nucleotide sequence identity and used in phylogenetic analysis if they represented OTUs from the iTag analysis (labeled in the trees "**contig-xxy MS-B SeqMan 96%**") . JGI IMG taxon ID 2015219002 represents an undermat metagenome dataset from a previous study (Klatt et al. 2013). Sequences of 16S rRNA were retrieved and used for phylogenetic analysis if they were related to sequences obtained in this study (labeled in the trees "**YNP15xxxxxx 16S rRNA [YNP15_xxxxx]**"). Additionally, 579 16S rRNA clone sequences obtained from a previous sample (Klatt et al. 2013) were assembled using 97% nt identity and used in phylogenetic analysis (labeled in the trees "**YNP15 clone SeqMan 97 contig YNP15_xxx**"). Phylogenetic analysis of rRNA genes was conducted using the software package ARB (www.arb-home.de, Ludwig et al., 2004) and the SILVA rRNA database release NR99_123 (www.arb-silva.de). Sequences were automatically aligned and the alignment refined manually. The sequences were added to the existing 1200_slv phylogenetic backbone treeof the SILVA_NR99_123 release without changing the overall topology to assess taxonomic affiliaton. Additional phylogenetic calculations using the Maximum Likelihood methods were conducted using the PHYML methods implemented in the ARB package, and bootstrapped by 100 replicates. Sequences of >1,000 bp length were used in phylogenetic calculations, and sequences with length <1,000bp were added to the phylogenetic tree using the Parsimony method without changing the tree topology. iTag sequences were not included in the phylogenetic analysis. In a few cases, sequences <500 bp (e.g., some OS type sequences) were added to the trees using the Parsimony method as mentioned above.
